# Genome-wide identification and functional prediction of tobacco lncRNAs responsive to root-knot nematode stress

**DOI:** 10.1371/journal.pone.0204506

**Published:** 2018-11-14

**Authors:** Xiaohui Li, Xuexia Xing, Shixiao Xu, Mingzhen Zhang, Yuan Wang, Hengyan Wu, Zhihao Sun, Zhaoguang Huo, Fang Chen, Tiezhao Yang

**Affiliations:** College of Tobacco, Henan Agricultural University, Zhengzhou city, Henan province, China; Dokuz Eylul Universitesi, TURKEY

## Abstract

Root-knot nematodes (RKNs, *Meloidogyne* spp.) are destructive plant parasites with a wide host range. They severely reduce crop quality and yield worldwide. Tobacco is a versatile model plant organism for studying RKNs-host interactions and a key plant material for molecular research. Long noncoding RNAs (lncRNAs) play critical roles in post transcriptional and transcriptional regulation in a wide range of biological pathways, especially plant development and stress response. In the present study, we obtained 5,206 high-confidence lncRNAs based on RNA sequencing data. Gene Ontology and Kyoto Encyclopedia of Genes and Genomes pathway analyses revealed that the target genes of these lncRNAs are mainly involved in plant biotic and abiotic stresses, plant hormone signal transduction, induced systemic resistance, plant-type hypersensitive response, plant-type cell wall organization or biogenesis. The 565 differentially expressed lncRNAs found to be involved in nematode stress response were validated by quantitative PCR using 15 randomly-selected lncRNA genes. Our study provides insights into the molecular mechanisms of RKNs-plant interactions that might help preventing nematode damages to crops.

## Introduction

Progress in RNA sequencing (RNA-seq) technologies has enabled the rapid exploration of protein-coding and noncoding RNAs in animal and plant genomes. Large-scale transcriptome studies in various species have identified many of the transcribed genomes. Nevertheless, only 10–20% of these sequences encode proteins [1]. Recently, it was found that long noncoding RNAs (lncRNAs) play important roles in eukaryotic gene regulation and are involved in several biological regulatory processes and resistance responses [2].

Generally, lncRNAs are transcripts ranging from 200 nucleotides (nt) to 100 kb in length, do not encode proteins, and are transcribed by RNA polymerase II [3]. Based on their genomic locations, lncRNAs are further classified into long intronic noncoding RNAs, long intergenic noncoding RNAs (lincRNAs), and long noncoding natural antisense transcripts (lncNATs). These are transcribed from the complementary DNA of their associated genes [4]. LncRNAs usually lack open reading frames (ORFs), are expressed at low levels, are not conserved among species, and often exhibit tissue- or cell-specific expression patterns [5]. Furthermore, lncRNAs participate in chromatin or histone modification, and transcriptional, post-transcriptional, and epigenetic regulation, among other processes, regulating gene expression [6].

In recent years, many lncRNAs have been identified in plants. In maize, 20,163 lincRNAs were identified by RNA-seq [7], 6,480 transcripts from Arabidopsis were classified as lncRNAs [8], and we obtained 125 putative stress-responsive lncRNAs in wheat [9]. Although numerous lncRNAs were identified, their functions still require further study.

Root-knot nematodes (RKNs; *Meloidogyne* spp.) are among the most destructive soil-borne crop pests worldwide. They have a wide host range and cause serious and costly yield losses in most cultivated plant species [9]. By parasitizing root systems and establishing permanent feeding sites, RKNs further induce giant cell or root-knot formation [10]. These deformations directly affect the uptake of water and nutrients, and therefore leaves become chlorotic, the shoots wilt, and the plants stunted. Overall, crop yield and quality are significantly reduced [11].

Currently, nematode infections are mainly controlled using chemical pesticides. Nevertheless, restrictions on the use of pest control products are increasing. Host resistance also plays an important role in nematode control, but conventional host resistance breeding depends on phenotypic selection and it is time-consuming and labor-intensive [12]. In contrast, using natural resistance sources and identifying novel resistance genes are effective nematode control methods [13].

Tobacco (*Nicotiana tabacum*), which is cultivated worldwide, is a host for several important nematode species. Generally, *N*. *tabacum* (2n = 4× = 48) is a complex allotetraploid with a ~4.5-Gb genome that evolved from interspecific hybridization. Tobacco is regarded as a versatile model plant for studying fundamental biological processes, functional genomics, and biotechnology applications [14].

With the development of next-generation sequencing technology, genome-wide transcriptome analysis may elucidate lncRNA-mediated gene regulation in tobacco-nematode interactions. However, it is still unknown if lncRNAs participate in the nematode defense network in tobacco. In the present study, we performed RNA-seq on parasite-responsive tobacco transcripts to isolate lncRNA genes expressed in tobacco roots that might be involved in RKN-host plant interaction. We obtained 5,206 high-confidence lncRNAs. Of these, 565 were found to be involved in nematode stress response. It is, therefore, necessary to identify novel lncRNAs of defense-related genes and investigate the signaling pathways in nematode defense response. Our study provides insights into the basal plant defense mechanisms of lncRNAs and nematode manipulation of host physiology [15].

## Materials and methods

### Plant materials and nematode inoculation

Two tobacco cultivars (G28 (resistant) and Long bohuang (susceptible)) were used in this study. Four- to six-week-old tobacco seedlings were transplanted into growth chambers set in a greenhouse with relative humidity (RH) of 80% and grown under a 16-h light (26°C)/8-h dark (18°C) cycle.

*Meloidogyne incognita* were harvested from roots of tomato plants (*Solanum lycopersicum* L. ‘Rutgers’) that had been inoculated with this nematode three months earlier, using a modified version of the method previously described by Nitao et al. [16]. The infected roots were collected at 10 days after infection (dpi). Infected and control roots were washed with sterile water, frozen in liquid nitrogen, and stored at -80°C.

### Total RNA extraction, RNA-seq library construction, and Illumina sequencing

Total RNA was extracted from roots with the Plant RNA Purification Reagent (Invitrogen, Carlsbad, CA, USA), according to the manufacturer’s instructions. Purified mRNA was used to construct cDNA libraries with the Illumina TruSeq^TM^ RNA Sample Prep Kit (Illumina, San Diego, CA, USA), following the manufacturer’s recommendations. Strand-specific sequencing was performed on the Illumina HiSeq 4000 system (paired-end 150-bp reads).

### Bioinformatics analysis to identify lncRNAs

Raw RNA-seq reads were filtered to remove low-quality reads and adaptor sequences (quality score, Q > 20). The resulting clean reads were mapped to the tobacco reference genome using the spliced read aligner TopHat [17]. We then compared the assemblies to the *N*. *tabacum* genome annotation using Cuffcompare. The lncRNA structure was optimized according to read distribution, paired-end information, and genome annotation. Assembled transcripts shorter than 200 bp and ORFs shorter than 100 residues were removed from the analysis, and the sequences of the remaining transcripts were compared with those for known noncoding RNAs (transfer RNA, ribosomal RNA, small nuclear RNA, small nucleolar RNA, precursor micro RNA, and pseudogenes) using CUFFLINKS [18]. Transcripts were compared with protein sequences deposited in the Swiss-Prot database using the basic local alignment search tool with a translated nucleotide query (BlastX) to eliminate protein-coding transcripts. Only multiple-exon transcripts with a number of fragments per kilobase of transcript per million mapped reads (FPKM) ≥ 0.5 or single-exon transcripts with FPKM ≥ 2 were retained. Coding Potential Calculator (CPC), RNA families database (Rfam), and Coding Non-Coding Index (CNCI)were used to filter the coding potentials of the remaining transcripts [19], which were regarded as reliably-expressed lncRNAs. Finally, 5,206 potential lncRNAs were used for further analyses.

### Quantitative reverse transcription PCR validation of lncRNAs

To validate the differentially expressed (DE) lncRNAs identified by RNA-seq, 15 lncRNAs from the resistant and susceptible tobacco lines were selected for quantitative reverse transcription PCR (qRT-PCR) validation. Three technical and three biological replicates were subject to qRT-PCR, using the SYBR Premix Ex TaqTM (Takara Bio Inc., Kyoto, Japan) and the primers listed in S6 Table. The amplification was performed under 5 min at 95°C followed by 40 cycles at 95°C for 10 s, 54°C for 20 s, and 72°C for 10 s. The relative expression of lncRNAs was calculated using the Ct (2^-ΔΔCt^) method.

### Target gene prediction and pathway enrichment analysis

Based on the genome location of the lncRNAs and protein-coding genes, we identified *cis*-acting lncRNAs target neighboring genes [20]. We searched for protein-coding genes 20-kb upstream and downstream of all identified lncRNAs and predicted their functional roles. Gene Ontology (GO) functional annotations of co-expressed genes were performed to predict lncRNA gene functions, and these were classified into biological process (BP), cellular component (CC), or molecular function (MF). Software KOBAS was used for testing the statistical enrichment of DE-lncRNA target genes in Kyoto Encyclopedia of Genes and Genomes (KEGG) pathways [21]. The GO terms and KEGG pathways with P < 0.05 were considered significantly enriched.

## Results

### Effects of nematode infection on the phenotypic traits of different tobacco genotypes

**Fig 1:**
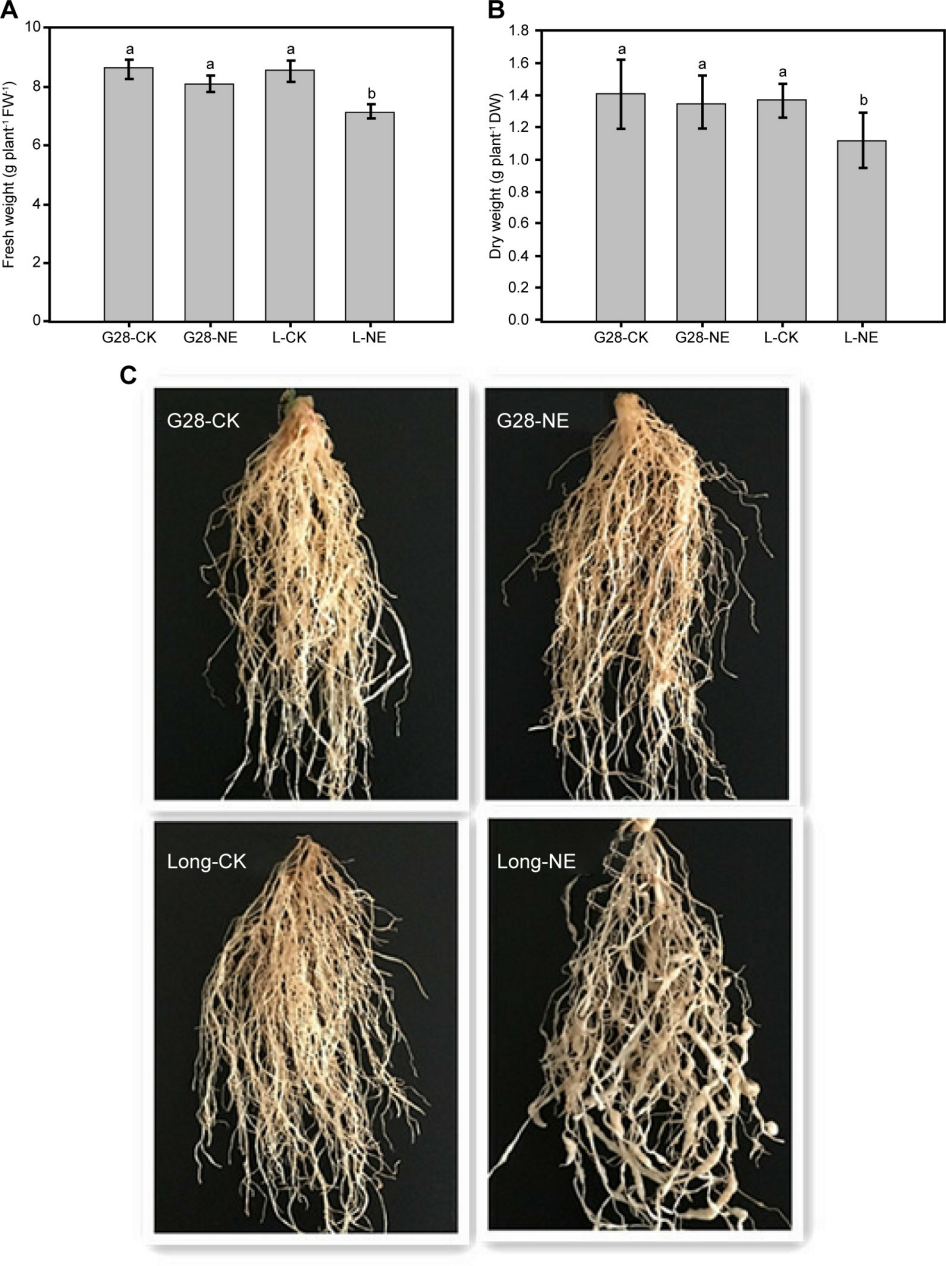
Phenotypic traits of G28 and Long bohuang. (A) fresh weight. (B) Dry weight. (C) Symptoms of nematode infection.

Two typical tobacco genotypes with contrasting responses to nematode infection were used in this study. The experiments demonstrated that the nematode-inoculated plants (NE) significantly differed in biomass from control plants (CK). Line G28 was only slightly affected in comparison to Long bohuang (Fig 1). The relative root fresh weight (NE/CK) was 97% for G28 and 84% for Long bohuang (Fig 1A, S1 Table) and the relative root dry weight was 96% for G28 and 82% for Long bohuang (Fig 1B, S1 Table). Fig 1C shows that the nematodes penetrated the roots of Long bohuang and then transformed injured root cells into giant cells or “knots”. These deformities severely impede root water and nutrient absorption in Long bohuang whereas the resistant cultivar G28 continued to grow and develop normally.

### Genome-wide identification of nematode-responsive lncRNAs in tobacco

An objective of this study was to compile a comprehensive lncRNAs catalog in *N*. *tabacum*. To this end, we obtained a high-quality, high-depth RNA-seq dataset from two tobacco genotypes subject to control and nematode inoculation treatments. We constructed four cDNA libraries and generated >129 million clean reads (average Q30 and GC were 94.05% and 44.90%, respectively) (Table 1). These data suggested that the sequencing quality and output were adequate for further analysis.

**Table 1:** Transcriptome sequencing results for nematode-inoculated (RKN) and control (CK) tobacco plants from resistant (G28) and susceptible (Long) lines.

Sample	Number of Raw Reads	Number of Clean Reads	Number of bases in Clean Reads	GC (%) in Clean Reads	Error rate (%)	Q20 (%)	Q30 (%)
G28CK	136 063 110	115 822 946	16 122 568 427	44.30	0.2164	97.78	93.87
G28RKN	167 474 876	146 012 906	20 414 656 427	44.09	0.2069	97.91	94.16
LongCK	111 997 142	88 937 804	12 413 682 252	45.87	0.2079	97.92	94.16
LongRKN	195 227 240	166 164 984	23 180 264 355	45.32	0.2118	97.87	94.03

**Fig 2:**
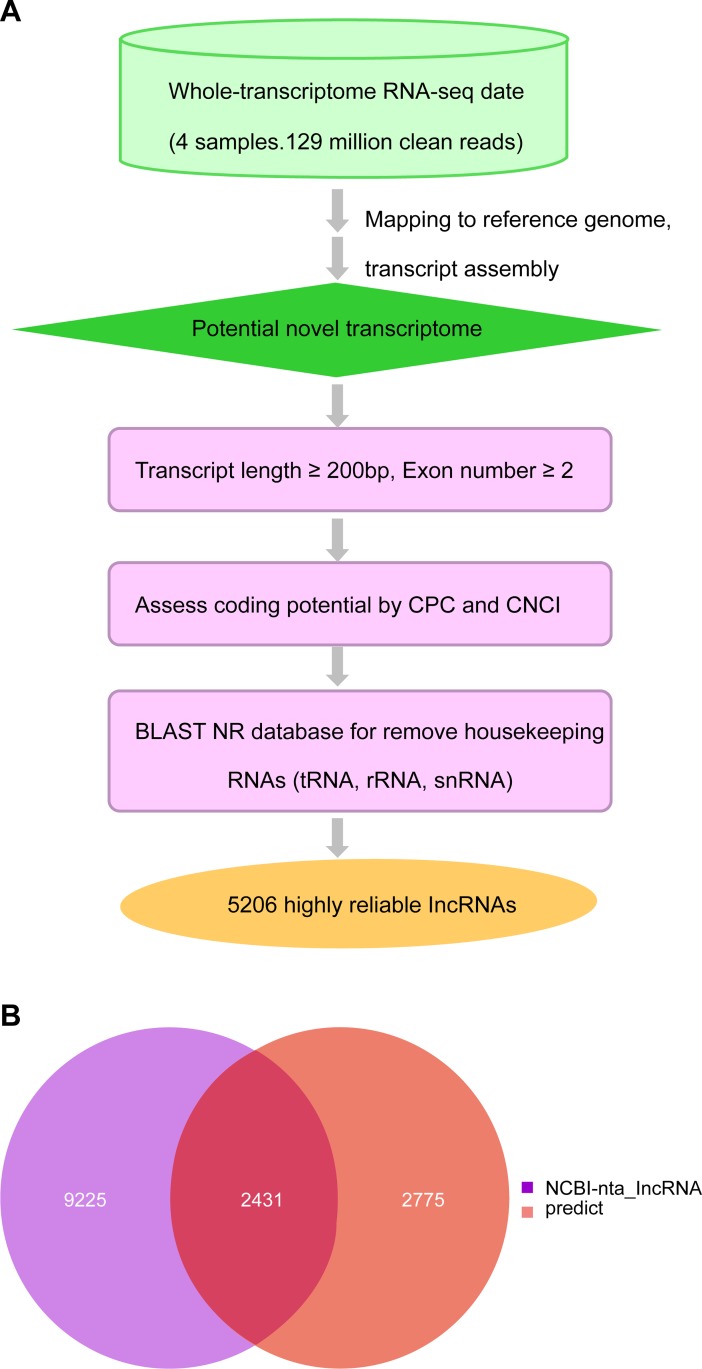
Identification of long noncoding RNAs (lncRNAs) in *Nicotiana tabacum*. (A) Bioinformatics pipeline for the systematic identification of lncRNAs in *N*. *tabacum*. (B) Venn diagram showing predicted lncRNAs and reference database lncRNAs.

We collected >516 million clean reads for lncRNAs identification using the Illumina HiSeq 4000 system. These reads were mapped to the tobacco reference genome followed by *de novo* transcriptome assembly using Cufflinks pipelines (Fig 2A), which allowed identifying 5,206 nematode-related lncRNAs in tobacco.

After assembling, annotating and filtering, we compared the predicted lncRNAs with those in the known reference database and presented them in a Venn diagram (Fig 2B). This diagram illustrates that 2,431 lncRNAs were common between predicted and known lncRNAs, and 9,225 and 2,775 lncRNAs were specifically expressed in the NCBI reference database, respectively.

### Characterization of tobacco lncRNAs

Previous studies have shown that plant and animal lncRNAs are shorter and harbor fewer exons than mRNAs [22]. We characterized the basic genomic features of the lncRNAs we obtained and compared them to the known features of tobacco protein-coding transcripts. The mean length of lncRNA transcripts (5,214 bp) was significantly greater than that of protein-coding transcripts (928 bp) (Part A in S1 Fig). The number of exons in tobacco lncRNAs ranged from 1 to >5, which is a significantly lower number than that of protein-coding transcripts. In fact, the average exon numbers were 27.3 for protein-coding transcripts and 7.3 for lncRNAs (Part B in S1 Fig). Most ORFs from lncRNAs were shorter than those from protein-coding transcripts, and the average lengths of ORFs were 596 nt for protein-coding transcripts and 134 nt for lncRNAs (Part C in S1 Fig). Taken together, these observations demonstrate that lncRNAs are relatively shorter and have fewer exons and shorter ORFs than mRNAs. Therefore, the lncRNAs found here are highly reliable.

### Differential expression of tobacco lncRNAs in response to nematode infection

**Fig 3:**
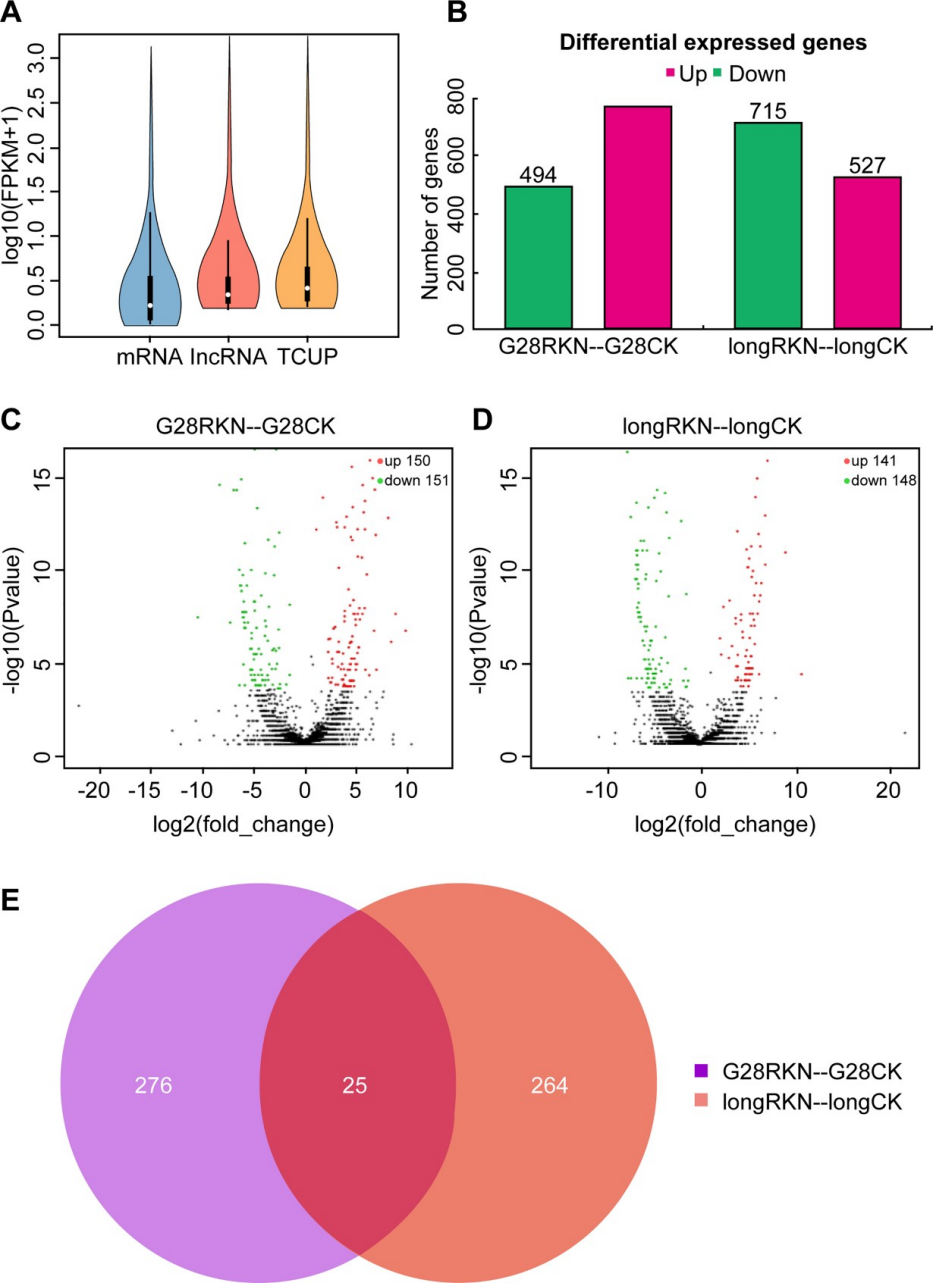
Differential expression of nematode response-related long non-coding RNAs (lncRNAs) in G28 and Long bohuang tobacco varieties. (A) Violin plot of mRNA, lncRNA, and TUCP expression levels. (B) Volcano plot of differentially expressed. (D,E) lncRNAs between the two different tobacco varieties. (C,D) Volcano plot of DE-lncRNAs in the two tobacco varieties. (E) Venn diagram showing DE-lncRNAs for nematode stress response.

Previous experiments have shown that, in mammals, the expression levels of lncRNAs are significantly lower than that of mRNAs [23]. We quantitatively determined the expression level of all screened transcripts, including mRNAs, lncRNAs, and transcripts of unknown coding potential (TUCP). In RNA-seq samples, FPKM values (i.e., transcript abundances) were higher for mRNAs than lncRNAs and TUCP. Therefore, lncRNAs were less transcribed and more conserved than mRNAs (Fig 3A).

In the present study, differentially expressed transcripts were defined based on P ≤ 0.05 and absolute log2 ratio ≥ 2. Among the 2,507 lncRNAs that were significantly and differentially expressed between the resistant and the susceptible tobacco varieties, 1,298 were upregulated and 1,209 were downregulated (Fig 3B). We identified significantly DE-lncRNAs in NE plants and compared them with CK plants. The results showed that 150 lncRNAs were upregulated and 151 lncRNAs were downregulated in G28 (Fig 3C, S2 Table). In contrast, 141 lncRNAs were upregulated and 148 lncRNAs were downregulated in Long bohuang (Fig 3D, S3 Table). We also investigated DE-lncRNAs expression in response to nematode stress. There were 276 DE-lncRNAs specifically expressed in G28 and 264 DE-lncRNAs specifically expressed in Long bohuang. Nevertheless, 25 lncRNAs were commonly expressed in both genotypes despite their different genetic backgrounds (Fig 3E). In summary, we identified 565 lncRNAs that might play significant roles in the response to nematode infection.

### Identification of nematode stress-responsive lncRNAs in tobacco

We identified the expression patterns of nematode-responsive lncRNAs in the four cDNA libraries. Twenty-five significantly and commonly expressed lncRNAs were identified, based on the log2 ratio ≥2 and P< 0.05 thresholds. The expression patterns of these commonly expressed lncRNAs were evaluated by systematic cluster analysis to explore similarities and compare their relationships in NE plants from the two genotypes (S2 Fig).

**Fig 4:**
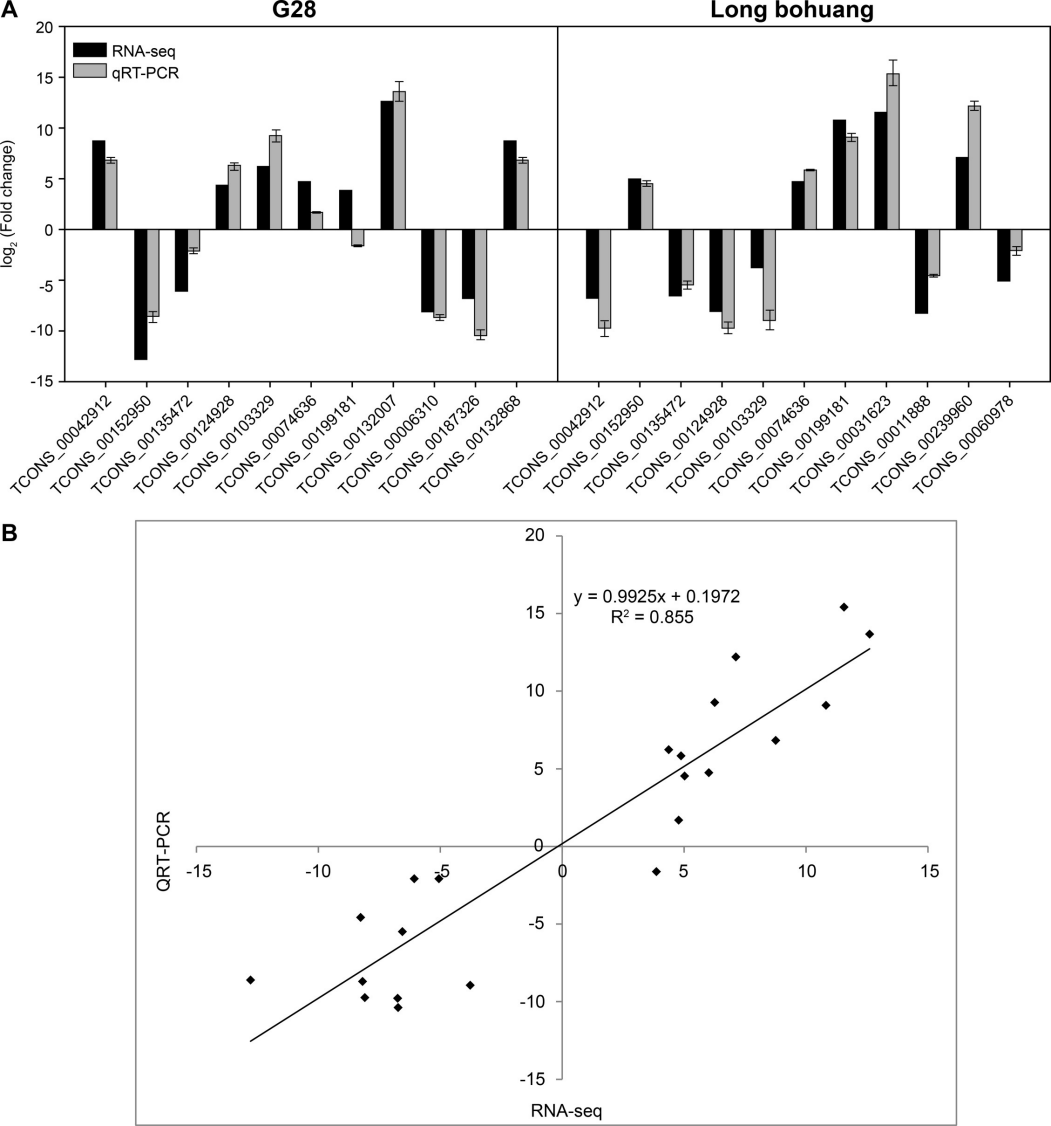
Validation of long non-coding RNAs expression patterns obtained by RNA sequencing (RNA-seq) using quantitative reverse transcription PCR (qRT-PCR). (A) Validation of RNA-seq results using qRT-PCR. (B) Correlation analysis of RNA-seq and qRT-PCR results.

The expression levels of tobacco lncRNAs obtained from RNA-seq were validated by qRT-PCR analysis using 15 randomly selected lncRNAs (Fig 4). The expression patterns of these lncRNAs were consistent with the results of RNA-seq, correlation coefficient (R^2^) as high as 0.855 (Fig 4A and 4B), indicating that the lncRNAs identified by RNA-seq were reliable.

### LncRNA target prediction, annotation, and enrichment analyses

LncRNAs usually play an important role in the regulation of their neighboring genes, functioning as *cis*-regulatory elements. To predict the functions of tobacco lncRNAs, we first predicted their potential targets in *cis*-regulatory relationships by searching for protein-coding genes 20kb upstream and downstream of all identified lncRNAs. The 565 nematode resistance-related lncRNAs that were transcribed close to 338 protein-coding genes were subject to GO analysis of *cis*-lncRNA targets to explore their potential functions. We found major GO terms associated with nematode stress response. For instance, the most enriched GO terms were ‘induced systemic resistance’ (GO:0009682), ‘plant-type hypersensitive response’ (GO:0009626), ‘plant-type cell wall organization or biogenesis’ (GO:0009505), ‘DNA binding transcription factor activity’ (GO:0003700), ‘peroxidase activity’ (GO:0004601), ‘response to oxidative stress’ (GO:0006979), and ‘hydrogen peroxide catabolic process’ (GO:0042744) (S4 and S5 Tables).

**Fig 5:**
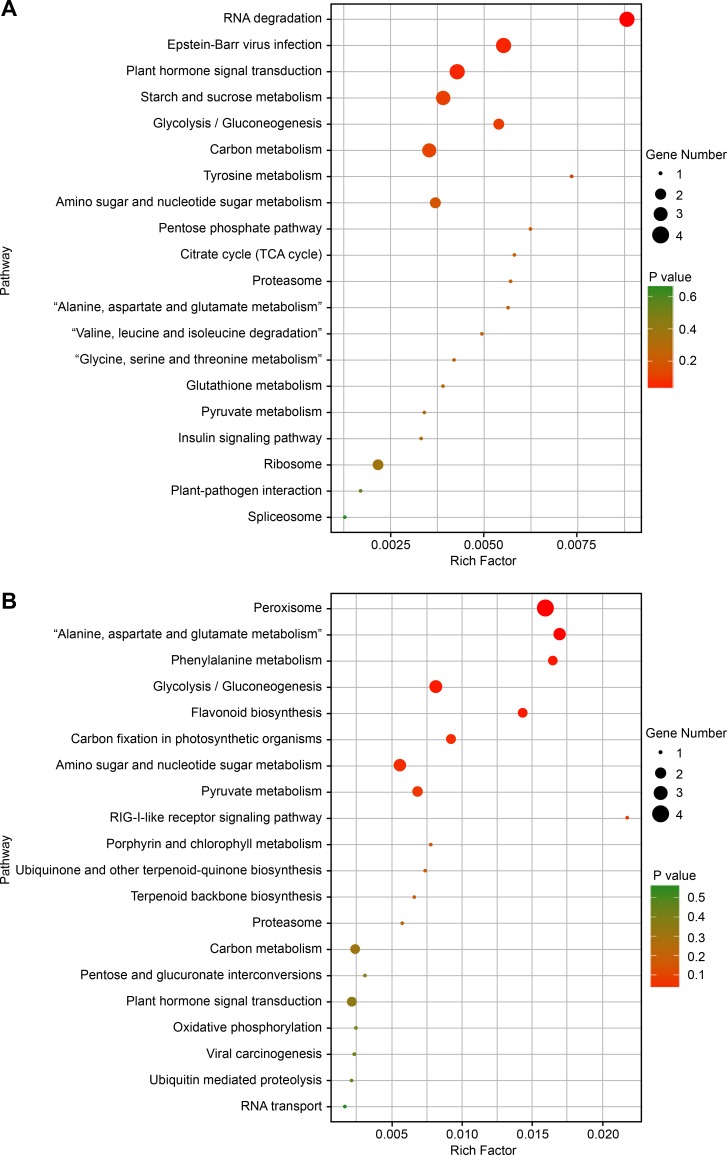
Enriched Kyoto Encyclopedia of Genes and Genomes (KEGG). (A) KEGG pathways enriched for the differentially expressed lncRNAs (DE-lncRNAs) correlated with differentially expressed genes (DEGs) in G28. (B) KEGG pathways enriched for DE-lncRNAs correlated with DEGs in Long bohuang. The color of the dots represents the P value, and its size represents the number of DEGs mapped to the reference pathways.

Based on the results of DE-lncRNAs, KOBAS software was used to analyze KEGG pathways. Among the 72 pathways responsive to nematode stress, those most enriched were ‘plant hormone signal transduction’, ‘carbon metabolism’, ‘phenylpropanoid biosynthesis’, ‘pentose phosphate’, ‘glutathione metabolism’, ‘plant-pathogen interaction’, ‘peroxisome’, and ‘phenylalanine metabolism’. These results suggested that lncRNAs might perform transcriptional regulation of genetic expression (Fig 5).

## Discussion

Plant-parasitizing nematodes cause serious damage to cultivated crops worldwide [13]. Chemical pesticides are frequently used to address this problem but they have adverse effects on the environment, humans, and wildlife [24]. Genetic improvement of crop resistance is a very good alternative to pest-control products, and genomic data is already available to produce such modifications in several crops [25]. Using nematode-resistant cultivars is a viable approach to reduce disease incidence. Tobacco is an ideal model plant for studying plant-parasitic nematodes and their pathogenesis [26]. Elucidating gene regulation and regulatory pathways will provide a molecular basis for nematode resistance research and enhance nematode-stress tolerance in tobacco. Thanks to advances in next-generation sequencing technology, lncRNAs have recently been revealed as regulatory mechanisms of various biological processes. Many novel lncRNAs transcripts participate in gene expression regulation and play crucial roles in development, reproduction, and biotic/abiotic stress responses.

Plants exhibit complex biochemical, physiological, and molecular responses to nematode stress. Nevertheless, the genome-wide lncRNAs responsive to nematode infection in tobacco have not yet been identified. In the present study, we used a strand-specific RNA-seq approach and genome-wide, systematically identified tobacco lncRNAs to find novel lncRNAs associated with nematode resistance. Strict screening criteria enabled us to identify 5,206 high-confidence lncRNAs. These identified lncRNAs unlock a new field in the investigation of novel regulatory pathways in plant-nematode interactions.

Most of these putative lncRNAs have 2–3 exons and a median length of 923 nt, and the expression levels of lncRNA transcripts were significantly lower than those of protein-coding genes. Hence, the overall features of tobacco lncRNAs agree with those determined for other organisms [27]. The lncRNAs are shorter than mRNAs, non-conserved in sequence, and can be spliced. Therefore, they might be a type of conserved genes undergoing rapid sequence evolution [28].

Long non-coding RNAs are endogenous RNAs that regulate gene expression and participate in developmental and physiological processes [29]. To understand the response mechanism of tobacco to nematode stress, we performed an integrated analysis of the lncRNA transcription levels. Among the 565 nematode stress-responsive lncRNAs identified, 25 were expressed in both genotypes; 276 DE-lncRNAs were specifically expressed in G28, and 264 DE-lncRNAs were specifically expressed in Long bohuang. Therefore, these lncRNAs might be important participants in the regulatory process of nematode resistance in tobacco.

The functions of certain lncRNAs have been confirmed but it is still unclear as to how lncRNAs participate in biological processes. Recent studies have shown that lncRNAs are involved in *cis*-regulation of target genes. Here, we identified a group of lncRNAs related to tobacco defense responses to nematode infection. Annotation analyses indicated that several genes involved in hormone signal transduction and secondary metabolic pathways were differentially expressed under nematode stress. Plants generally respond to nematode invasion by differentially expressing genes involved inmetabolism, hormonal signaling transduction, cell wall architecture, stress, and defense responses [30]. Genes involved in such pathways were differentially expressed in the present study. Therefore, tobacco lncRNAs might play important roles in host resistance or susceptibility to nematode infection.

In accordance with previous observations, several genes involved in cell wall organization remodeling were differentially expressed in tobacco after nematode infection. The plant cell wall is composed of pectin, hemicelluloses, andcellulose microfibrils, representinga structural barrier to pathogen infection. Endoparasitic nematodes have evolved sophisticated resistance mechanisms to break this barrier and secrete cell wall-degrading or -modifying enzymes (CWD/MEs) into host plant roots [31]. After the initiation of feeding, the expression of CWD/MEs decreased in nematodes, which induced the remodeling of cell walls for the formation of feeding cells [32]. Our results showed that the expression of cell wall biosynthesis-related genes was upregulated, which might be a plant defense strategy against migratory nematode infection in the roots.

Recent evidence demonstrated that reactive oxygen species (ROS) network pathways play crucial roles in pathogen resistance signal transduction [33]. These compounds are rapidly produced in plants after infection and may prevent the pathogen from entering the cell and/or induce resistance-gene expression [34]. In host plant-nematode interactions, the nematode produces a series of peroxiredoxins, such as catalase, ascorbate peroxidase, peroxidase, phenylalanine ammonia-lyase, and superoxide dismutase to evade plant defense responses. In ROS scavenging, significant induction of these enzymes can minimize ROS accumulation and reduce cell membrane injury in the early steps of host–nematode interactions [35]. Taken together, the ROS burst negatively affects nematode infection and then triggers hypersensitive reaction in an attempt to block nematode development.

Plant hormones are involved in plants’ response against nematode invasion [36]. Nematode invasion breaks hormonal homeostasis, due to the interaction between active manipulation of nematode effectors secreted in the plant organism, which activates defense reaction and reduces pathogen infection [37]. In our transcriptome analysis, many plant hormone-related genes involved in ethylene (ET), jasmonic acid (JA), salicylic acid (SA), abscisic acid, gibberellic acid, and auxin signaling pathways were identified. Genes encoding pathogenesis-related 1 protein were upregulated by SA in Arabidopsis-nematode interaction [38], and Nahar's research indicated that the JA pathway mediated by ET, is a crucial player in systemically-induced defense against nematodesin rice [39]. The BCL2-antagonist/killer 1 (BAK1) protein is involved in brassinosteroid (BR) signaling, and activation of the BR pathway might improve *BAK1* gene expression in nematode-infected rice roots, which overcome root defense [39]. Auxin manipulation is known to be a key process during the initiation and development of the feeding sites of sedentary plant nematodes [40]. Genes involved in the pathways mentioned above were differentially regulated during resistance or susceptible responses in tobacco-nematode interactions suggesting that hormone biosynthesis-related genes might be involved in the resistance response of tobacco to nematode infection.

Transcription factors (TFs) play important roles in the host plant responses to nematode infection. Many target TFs negatively or positively respond to nematode stress. These include SPL, MYB, CKX, ARF, DCL1, NAM, WRKY, TCP, NAC, ZIP, NFYA, and TOE. These TFs activate miRNAs, regulate gene expression, and enhance plant nematode stress resistance. The WRKY factors are main players of the innate immune system of plants and widely participate in host plant responses to nematode invasion. Some studies indicated that WRKY11 and WRKY17 are positive regulators of nematode defense and negatively regulated gene expression in Arabidopsis roots [41]. In addition, WRKY2 is a pathogen-inducible defense-signaling that is preferentially induced during the incompatible interactions of pepper with pathogens [42]. Thus, differentially expressed TFs are closely related to their functions and might activate or repress the expression of down-stream genes that respond to nematode infection.

In conclusion, our study is the first to identify and characterize, on a genome-wide scale, lncRNAs in tobacco inoculated with nematodes. We identified 565 nematode-responsive lncRNAs in two different tobacco genotypes. This study provides deep and new insights into the complex molecular mechanisms involved in plant-nematode interactions. To elucidate these processes and the biological functions of lncRNAs in model plants, further experimental validation is needed using overexpressed or transgenic RNA interference.

## Supporting information

S1 Fig Characteristics of long non-coding RNAs (lncRNAs) in *Nicotiana tabacum*. (A) Length density distribution of lncRNAs and mRNAs. (B) Exon number distribution per transcript of lncRNAs and mRNAs. (C) Open reading frame protein length distribution of lncRNAs and mRNAs.
(TIF)

S2 Fig Hierarchical clustering analysis of commonly expressed long non-coding RNAs (lncRNAs) under root-knot nematode infection and control conditions. The sample and treatments are displayed below each column. Genes are indicated by different colors. Relative levels of expression are shown using a color gradient from low (green) to high (red).
(PNG)

S1 Table Phenotypic traits of G28 and Long bohuang between different treatments. (DOC)

S2 Table Significantly differentially expressed long non-coding RNAs in G28. (DOC)

S3 Table Significantly differentially expressed long non-coding RNAs in Long bohuang. (DOC)

S4 Table Gene ontology classification of target transcripts for all differentially expressed long non-coding RNAs in G28. (DOCX)

S5 Table Gene ontology classification of target transcripts for all differentially expressed long non-coding RNAs in Long bohuang. (DOCX)

S6 Table Quantitative reverse transcription PCR primers for RNA sequencing results validation. (DOCX)
