# Supplementary figures and images for: Genome-wide identification and functional prediction of tobacco lncRNAs responsive to root-knot nematode stress
Source: PLoS One. 2018 Nov 14;13(11):e0204506. doi: 10.1371/journal.pone.0204506 (PMC6235259; doi:10.1371/journal.pone.0204506)

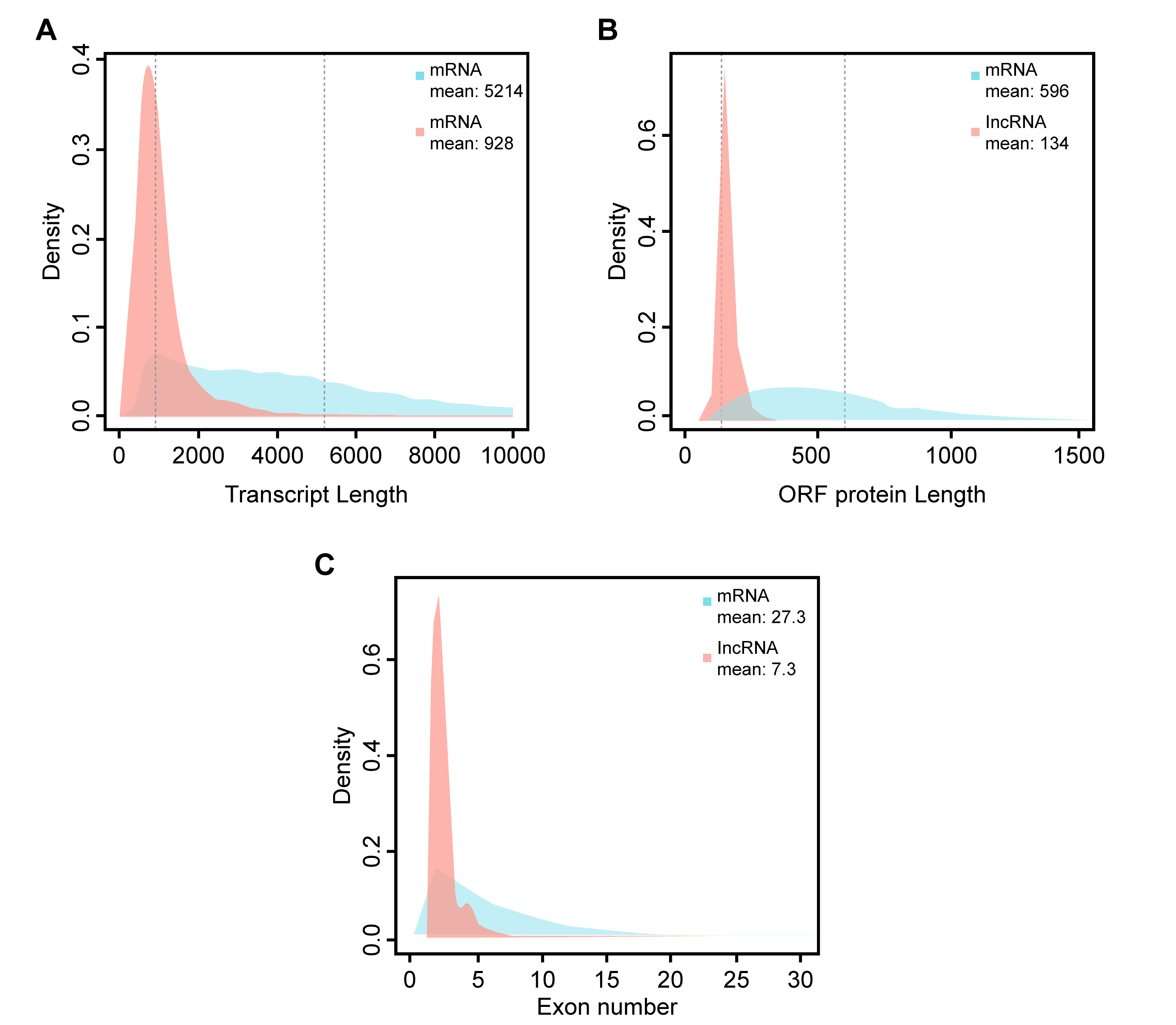

Supplement: S1 Fig — (A) Length density distribution of lncRNAs and mRNAs. (B) Exon number distribution per transcript of lncRNAs and mRNAs. (C) Open reading frame protein length distribution of lncRNAs and mRNAs. (TIF) [file pone.0204506.s001.tif]

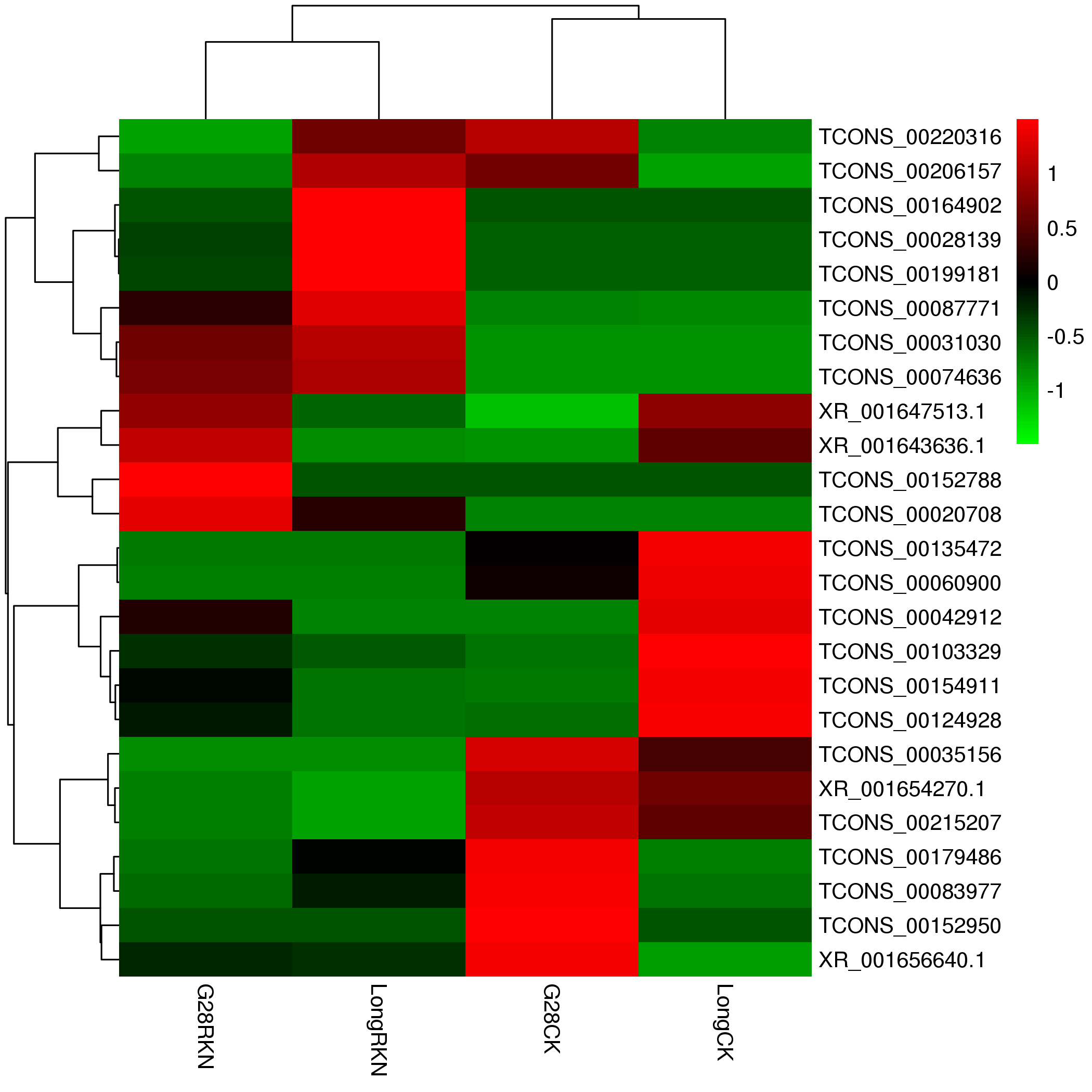

Supplement: S2 Fig — The sample and treatments are displayed below each column. Genes are indicated by different colors. Relative levels of expression are shown using a color gradient from low (green) to high (red). (PNG) [file pone.0204506.s002.png]
