# Supplementary material for: Genome-wide identification and functional prediction of tobacco lncRNAs responsive to root-knot nematode stress
Source: PLoS One. 2018 Nov 14;13(11):e0204506. doi: 10.1371/journal.pone.0204506 (PMC6235259; doi:10.1371/journal.pone.0204506)
Supplement: S1 Table — (DOC) [file pone.0204506.s003.doc]

**S1 Table. phenotypic traits of G28 and Long bohuang between different treatments**

| Index | G28-CK | G28-NE | C-CK | C-NE |
| --- | --- | --- | --- | --- |
| fresh weight | 8.52±0.12 | 8.24±0.11 | 8.52±0.35 | 7.15±0.24 |
| dry weight | 1.41±0.21 | 1.35±0.16 | 1.37±0.10 | 1.12±0.17 |
