# Supplementary material for: Genome-wide identification and functional prediction of tobacco lncRNAs responsive to root-knot nematode stress
Source: PLoS One. 2018 Nov 14;13(11):e0204506. doi: 10.1371/journal.pone.0204506 (PMC6235259; doi:10.1371/journal.pone.0204506)
Supplement: S2 Table — (DOC) [file pone.0204506.s004.doc]

**S2 Table. Significantly differentially expressed lncRNAs in G28.**

| Transcript | Gene | Type | G28CK | G28RKN | log2 (foldchange) | P-value | Q-value |
| --- | --- | --- | --- | --- | --- | --- | --- |
| XR_001647192.1 | LOC107782282 | lncRNA | 40.651394 | 156.334763 | 1.94326 | 0 | 0 |
| XR_001644615.1 | LOC107770431 | lncRNA | 201.006271 | 3.459651 | -5.86047 | 0 | 0 |
| XR_001645221.1 | LOC107773033 | lncRNA | 0.516945 | 57.209454 | 6.7901 | 0 | 0 |
| XR_001653677.1 | LOC107810583 | lncRNA | 154.702515 | 0.498864 | -8.27663 | 0 | 0 |
| TCONS_00152789 | XLOC_078470 | lncRNA | 1.039523 | 1419.702637 | 10.4155 | 0 | 0 |
| TCONS_00152788 | XLOC_078469 | lncRNA | 0.917314 | 6621.360352 | 12.8174 | 0 | 0 |
| TCONS_00171242 | XLOC_087798 | lncRNA | 795.179626 | 0.538612 | -10.5278 | 0 | 0 |
| TCONS_00171245 | XLOC_087795 | lncRNA | 1.322845 | 818.342346 | 9.27292 | 0 | 0 |
| TCONS_00171246 | XLOC_087798 | lncRNA | 3.927917 | 319.101318 | 6.34411 | 0 | 0 |
| TCONS_00206257 | XLOC_105654 | lncRNA | 140.97612 | 0.850806 | -7.3724 | 0 | 0 |
| TCONS_00239202 | XLOC_122384 | lncRNA | 132.064011 | 0.466728 | -8.14444 | 0 | 0 |
| TCONS_00202050 | XLOC_103498 | lncRNA | 64.75573 | 0.527755 | -6.939 | 0 | 0 |
| TCONS_00177339 | XLOC_090875 | lncRNA | 0.690713 | 136.050873 | 7.62184 | 0 | 0 |
| TCONS_00236176 | XLOC_120826 | lncRNA | 26962.62695 | 1.679423 | -13.9707 | 0 | 0 |
| TCONS_00035478 | XLOC_018262 | lncRNA | 0.665165 | 59.702007 | 6.48792 | 0 | 0 |
| TCONS_00177420 | XLOC_090906 | lncRNA | 1.516086 | 59.589142 | 5.29663 | 0 | 0 |
| TCONS_00123298 | XLOC_063273 | lncRNA | 5612.731445 | 0.888265 | -12.6254 | 0 | 0 |
| TCONS_00075694 | XLOC_038921 | lncRNA | 64.23243 | 0.426868 | -7.23337 | 0 | 0 |
| TCONS_00154065 | XLOC_079138 | lncRNA | 1598.074097 | 0.503615 | -11.6317 | 0 | 0 |
| TCONS_00035156 | XLOC_018095 | lncRNA | 62.016087 | 0.427301 | -7.18125 | 0 | 0 |
| TCONS_00224126 | XLOC_114714 | lncRNA | 0.576575 | 79.44175 | 7.10625 | 0 | 0 |
| TCONS_00085593 | XLOC_043908 | lncRNA | 393.927979 | 1.133085 | -8.44153 | 0 | 0 |
| TCONS_00093836 | XLOC_048106 | lncRNA | 0.493313 | 231.069351 | 8.87161 | 0 | 0 |
| TCONS_00042908 | XLOC_022090 | lncRNA | 0.52827 | 228.012924 | 8.75362 | 0 | 0 |
| TCONS_00132007 | XLOC_067967 | lncRNA | 0.555371 | 3461.124023 | 12.6055 | 0 | 0 |
| TCONS_00223689 | XLOC_114481 | lncRNA | 547.733215 | 0 | #NAME? | 0 | 0 |
| TCONS_00240161 | XLOC_122856 | lncRNA | 571.783569 | 7.935166 | -6.17106 | 0 | 0 |
| TCONS_00028139 | XLOC_014484 | lncRNA | 0.512528 | 66.140488 | 7.01176 | 0 | 0 |
| TCONS_00141082 | XLOC_072554 | lncRNA | 2.527632 | 711.11377 | 8.13615 | 0 | 0 |
| TCONS_00161041 | XLOC_082713 | lncRNA | 0.646129 | 760.520386 | 10.2009 | 0 | 0 |
| TCONS_00044212 | XLOC_022710 | lncRNA | 0.646969 | 769.693298 | 10.2164 | 0 | 0 |
| TCONS_00006310 | XLOC_003284 | lncRNA | 143.354202 | 0.489841 | -8.19305 | 0 | 0 |
| TCONS_00114046 | XLOC_058531 | lncRNA | 1.368633 | 61.064457 | 5.47953 | 0 | 0 |
| TCONS_00089759 | XLOC_046018 | lncRNA | 1.925404 | 297.534088 | 7.27175 | 0 | 0 |
| TCONS_00031675 | XLOC_016308 | lncRNA | 1.686833 | 73.096413 | 5.43741 | 0 | 0 |
| TCONS_00000813 | XLOC_000435 | lncRNA | 0.882643 | 317.926361 | 8.49265 | 0 | 0 |
| TCONS_00132986 | XLOC_068447 | lncRNA | 73.553329 | 0.849992 | -6.4352 | 0 | 0 |
| TCONS_00124582 | XLOC_064053 | lncRNA | 110.454773 | 1.730083 | -5.99647 | 0 | 0 |
| TCONS_00143432 | XLOC_073705 | lncRNA | 878.432922 | 0.412412 | -11.0566 | 0 | 0 |
| TCONS_00037356 | XLOC_019233 | lncRNA | 204.477921 | 0.609505 | -8.39009 | 0 | 0 |
| TCONS_00037357 | XLOC_019234 | lncRNA | 1.096502 | 109.077431 | 6.6363 | 0 | 0 |
| TCONS_00231137 | XLOC_118228 | lncRNA | 88.645393 | 0.642032 | -7.10926 | 0 | 0 |
| TCONS_00179486 | XLOC_092022 | lncRNA | 117.450569 | 2.60975 | -5.492 | 0 | 0 |
| TCONS_00100933 | XLOC_051878 | lncRNA | 3.026561 | 116.733574 | 5.2694 | 0 | 0 |
| TCONS_00094130 | XLOC_048255 | lncRNA | 4.673831 | 74.689987 | 3.99824 | 0 | 0 |
| TCONS_00029849 | XLOC_015361 | lncRNA | 0.598911 | 271.126892 | 8.82241 | 0 | 0 |
| TCONS_00124212 | XLOC_063827 | lncRNA | 230.474289 | 5.950153 | -5.27554 | 0 | 0 |
| TCONS_00000802 | XLOC_000440 | lncRNA | 0.85348 | 521.051147 | 9.25385 | 0 | 0 |
| TCONS_00000805 | XLOC_000443 | lncRNA | 222.151825 | 0.370632 | -9.22734 | 0 | 0 |
| TCONS_00157324 | XLOC_080861 | lncRNA | 113.657433 | 0.479409 | -7.88922 | 0 | 0 |
| TCONS_00152950 | XLOC_078549 | lncRNA | 3634.400635 | 0.516029 | -12.782 | 0 | 0 |
| TCONS_00143555 | XLOC_073772 | lncRNA | 237.283066 | 2.391217 | -6.63272 | 0 | 0 |
| TCONS_00060703 | XLOC_031159 | lncRNA | 4610.332031 | 0.730162 | -12.6244 | 0 | 0 |
| TCONS_00042216 | XLOC_021733 | lncRNA | 169.550873 | 0.525279 | -8.33442 | 0 | 0 |
| TCONS_00066203 | XLOC_033960 | lncRNA | 280.54834 | 0.49268 | -9.15338 | 0 | 0 |
| TCONS_00207860 | XLOC_106451 | lncRNA | 0.566193 | 56.584751 | 6.64298 | 0 | 0 |
| TCONS_00093284 | XLOC_047810 | lncRNA | 8242.511719 | 0.374849 | -14.4245 | 0 | 0 |
| TCONS_00095156 | XLOC_048812 | lncRNA | 1121.967163 | 1.496048 | -9.55066 | 0 | 0 |
| TCONS_00127097 | XLOC_065490 | lncRNA | 5.492767 | 5156.469727 | 9.87463 | 0 | 0 |
| TCONS_00125086 | XLOC_064356 | lncRNA | 1.941898 | 4325.737305 | 11.1213 | 0 | 0 |
| TCONS_00125084 | XLOC_064356 | lncRNA | 5609.113281 | 0 | #NAME? | 0 | 0 |
| TCONS_00054087 | XLOC_027683 | lncRNA | 266.758453 | 1.393445 | -7.58073 | 0 | 0 |
| TCONS_00015150 | XLOC_007828 | lncRNA | 415.128967 | 6.473254 | -6.00292 | 0 | 0 |
| TCONS_00034682 | XLOC_017848 | lncRNA | 86.951714 | 2.895044 | -4.90856 | 0 | 0 |
| TCONS_00078279 | XLOC_040204 | lncRNA | 1.411261 | 936.064087 | 9.37348 | 0 | 0 |
| TCONS_00026919 | XLOC_013875 | lncRNA | 0.680962 | 199.535217 | 8.19485 | 0 | 0 |
| TCONS_00110501 | XLOC_056665 | lncRNA | 0.309897 | 107.558212 | 8.43911 | 0 | 0 |
| TCONS_00000899 | XLOC_000488 | lncRNA | 296.548553 | 0.409664 | -9.49961 | 0 | 0 |
| TCONS_00187884 | XLOC_096272 | lncRNA | 751.902771 | 0 | #NAME? | 0 | 0 |
| TCONS_00113594 | XLOC_058288 | lncRNA | 261.805328 | 3.507026 | -6.2221 | 0 | 0 |
| TCONS_00025326 | XLOC_013081 | lncRNA | 718.427368 | 0.89549 | -9.64795 | 0 | 0 |
| TCONS_00025327 | XLOC_013083 | lncRNA | 1511.957886 | 0.696275 | -11.0845 | 0 | 0 |
| TCONS_00054093 | XLOC_027683 | lncRNA | 0 | 1497.234863 | inf | 0 | 0 |
| TCONS_00017157 | XLOC_008885 | lncRNA | 77.527237 | 1.471739 | -5.71911 | 0 | 0 |
| TCONS_00187326 | XLOC_095994 | lncRNA | 68.651512 | 0.643489 | -6.73723 | 0 | 0 |
| TCONS_00131227 | XLOC_067578 | lncRNA | 3.50366 | 89.151642 | 4.66933 | 0 | 0 |
| TCONS_00210116 | XLOC_107623 | lncRNA | 47.510929 | 567.894165 | 3.57929 | 0 | 0 |
| TCONS_00190600 | XLOC_097620 | lncRNA | 111.752289 | 0.598526 | -7.54467 | 0 | 0 |
| TCONS_00007852 | XLOC_004079 | lncRNA | 1241.081909 | 1.182785 | -10.0352 | 0 | 0 |
| TCONS_00063997 | XLOC_032810 | lncRNA | 1.05378 | 69.334045 | 6.03992 | 0 | 0 |
| TCONS_00167751 | XLOC_086122 | lncRNA | 0.564929 | 207.76683 | 8.52268 | 0 | 0 |
| TCONS_00074810 | XLOC_038457 | lncRNA | 68.843361 | 1.468819 | -5.55059 | 0 | 0 |
| TCONS_00110468 | XLOC_056651 | lncRNA | 3.011226 | 139.325027 | 5.53196 | 0 | 0 |
| TCONS_00072367 | XLOC_037135 | lncRNA | 179.346375 | 4.334438 | -5.37076 | 0 | 0 |
| XR_001646007.1 | LOC107776601 | lncRNA | 98.573082 | 13.922625 | -2.82376 | 1.11E-16 | 3.08E-14 |
| TCONS_00104847 | XLOC_053878 | lncRNA | 66.316833 | 2.203936 | -4.91122 | 1.11E-16 | 3.08E-14 |
| TCONS_00127769 | XLOC_065875 | lncRNA | 0.645331 | 51.50444 | 6.31851 | 4.44E-16 | 1.19E-13 |
| TCONS_00129565 | XLOC_066783 | lncRNA | 2.481456 | 58.232838 | 4.55257 | 9.99E-16 | 2.64E-13 |
| TCONS_00000905 | XLOC_000498 | lncRNA | 0.499218 | 48.046402 | 6.58861 | 4.11E-15 | 1.06E-12 |
| TCONS_00123698 | XLOC_063515 | lncRNA | 51.820183 | 0.690905 | -6.22888 | 4.77E-15 | 1.21E-12 |
| TCONS_00181120 | XLOC_092876 | lncRNA | 50.527267 | 0.155379 | -8.34513 | 9.33E-15 | 2.34E-12 |
| TCONS_00000114 | XLOC_000054 | lncRNA | 1.043513 | 51.154564 | 5.61534 | 9.66E-15 | 2.41E-12 |
| TCONS_00078092 | XLOC_040115 | lncRNA | 0.41579 | 46.27705 | 6.7983 | 1.70E-14 | 4.17E-12 |
| TCONS_00222289 | XLOC_113768 | lncRNA | 49.409527 | 0.394408 | -6.96896 | 1.80E-14 | 4.37E-12 |
| TCONS_00233460 | XLOC_119420 | lncRNA | 49.392174 | 0.484627 | -6.67126 | 1.80E-14 | 4.37E-12 |
| TCONS_00000777 | XLOC_000414 | lncRNA | 39.682358 | 132.002777 | 1.734 | 4.62E-14 | 1.07E-11 |
| TCONS_00031030 | XLOC_015950 | lncRNA | 0.78065 | 44.549068 | 5.83458 | 6.95E-14 | 1.60E-11 |
| TCONS_00033267 | XLOC_017154 | lncRNA | 1.91205 | 47.638546 | 4.63894 | 1.51E-13 | 3.40E-11 |
| TCONS_00083977 | XLOC_043104 | lncRNA | 50.768948 | 1.998323 | -4.66708 | 1.70E-13 | 3.80E-11 |
| TCONS_00207126 | XLOC_106120 | lncRNA | 0.817957 | 42.923939 | 5.71361 | 2.85E-13 | 6.27E-11 |
| TCONS_00216428 | XLOC_110773 | lncRNA | 38.828362 | 0 | #NAME? | 4.58E-13 | 9.87E-11 |
| TCONS_00018671 | XLOC_009670 | lncRNA | 3.194321 | 52.52562 | 4.03944 | 4.95E-13 | 1.07E-10 |
| TCONS_00000801 | XLOC_000439 | lncRNA | 0.150223 | 41.235569 | 8.10064 | 5.76E-13 | 1.22E-10 |
| TCONS_00074610 | XLOC_038354 | lncRNA | 7.576913 | 62.608967 | 3.04669 | 9.86E-13 | 2.06E-10 |
| XR_001647513.1 | LOC107783772 | lncRNA | 7.088231 | 61.544022 | 3.11812 | 1.81E-12 | 3.65E-10 |
| TCONS_00199181 | XLOC_102013 | lncRNA | 3.516937 | 50.969189 | 3.85723 | 1.83E-12 | 3.69E-10 |
| TCONS_00167111 | XLOC_085766 | lncRNA | 1.25385 | 43.318531 | 5.11055 | 2.34E-12 | 4.67E-10 |
| TCONS_00160811 | XLOC_082584 | lncRNA | 0.690556 | 39.385406 | 5.83376 | 2.36E-12 | 4.69E-10 |
| TCONS_00098317 | XLOC_050484 | lncRNA | 105.271584 | 224.387817 | 1.09188 | 2.41E-12 | 4.78E-10 |
| XR_001654270.1 | LOC107813192 | lncRNA | 79.277634 | 13.648949 | -2.53812 | 3.57E-12 | 6.88E-10 |
| TCONS_00038755 | XLOC_019951 | lncRNA | 0.322364 | 38.171917 | 6.88768 | 4.77E-12 | 9.17E-10 |
| TCONS_00242052 | XLOC_123835 | lncRNA | 2.073613 | 45.758862 | 4.46383 | 6.21E-12 | 1.19E-09 |
| TCONS_00077325 | XLOC_039769 | lncRNA | 54.807152 | 4.512192 | -3.60246 | 8.71E-12 | 1.65E-09 |
| TCONS_00088925 | XLOC_045614 | lncRNA | 1.69261 | 41.108105 | 4.6021 | 9.18E-12 | 1.72E-09 |
| TCONS_00235739 | XLOC_120607 | lncRNA | 39.823898 | 0.680182 | -5.87157 | 1.34E-11 | 2.44E-09 |
| TCONS_00085176 | XLOC_043701 | lncRNA | 61.90731 | 7.834269 | -2.98224 | 2.01E-11 | 3.61E-09 |
| TCONS_00020990 | XLOC_010821 | lncRNA | 1.103973 | 38.715973 | 5.13215 | 7.09E-11 | 1.21E-08 |
| TCONS_00107943 | XLOC_055371 | lncRNA | 0.746447 | 34.655388 | 5.5369 | 7.94E-11 | 1.34E-08 |
| TCONS_00230774 | XLOC_118072 | lncRNA | 4.614618 | 45.031605 | 3.28665 | 2.81E-10 | 4.55E-08 |
| TCONS_00198613 | XLOC_101702 | lncRNA | 34.070793 | 0.396892 | -6.42365 | 3.64E-10 | 5.85E-08 |
| TCONS_00217623 | XLOC_111362 | lncRNA | 38.390488 | 1.002924 | -5.25846 | 3.72E-10 | 5.95E-08 |
| TCONS_00017594 | XLOC_009114 | lncRNA | 44.690094 | 3.032752 | -3.88126 | 5.62E-10 | 8.90E-08 |
| TCONS_00132868 | XLOC_068376 | lncRNA | 0.481366 | 31.013821 | 6.00963 | 6.53E-10 | 1.03E-07 |
| TCONS_00063499 | XLOC_032555 | lncRNA | 33.697563 | 0.498865 | -6.07785 | 7.03E-10 | 1.10E-07 |
| XR_001646009.1 | LOC107776605 | lncRNA | 52.825287 | 6.510698 | -3.02034 | 7.20E-10 | 1.12E-07 |
| TCONS_00242367 | XLOC_123992 | lncRNA | 31.183821 | 0.386509 | -6.33415 | 2.62E-09 | 3.84E-07 |
| TCONS_00132965 | XLOC_068434 | lncRNA | 31.307642 | 0.606437 | -5.69001 | 2.62E-09 | 3.84E-07 |
| TCONS_00028195 | XLOC_014525 | lncRNA | 31.21043 | 0.410149 | -6.24974 | 2.62E-09 | 3.84E-07 |
| TCONS_00147245 | XLOC_075647 | lncRNA | 1.737255 | 32.387669 | 4.22056 | 4.13E-09 | 5.95E-07 |
| TCONS_00233288 | XLOC_119325 | lncRNA | 30.760147 | 0.420331 | -6.19339 | 5.06E-09 | 7.20E-07 |
| TCONS_00094894 | XLOC_048671 | lncRNA | 30.139347 | 0.40561 | -6.21541 | 5.06E-09 | 7.20E-07 |
| TCONS_00072503 | XLOC_037205 | lncRNA | 33.167999 | 1.699609 | -4.28652 | 8.86E-09 | 1.23E-06 |
| XR_001643636.1 | LOC107766229 | lncRNA | 0 | 22.61371 | inf | 9.69E-09 | 1.33E-06 |
| TCONS_00114300 | XLOC_058660 | lncRNA | 1.143291 | 30.138527 | 4.72034 | 1.59E-08 | 2.15E-06 |
| TCONS_00220316 | XLOC_112742 | lncRNA | 28.196718 | 0.945163 | -4.89882 | 1.88E-08 | 2.51E-06 |
| TCONS_00140195 | XLOC_072138 | lncRNA | 28.497616 | 0.891415 | -4.9986 | 1.88E-08 | 2.51E-06 |
| TCONS_00060861 | XLOC_031235 | lncRNA | 28.04814 | 0.442476 | -5.98616 | 1.88E-08 | 2.51E-06 |
| TCONS_00002105 | XLOC_001083 | lncRNA | 22.057505 | 0 | #NAME? | 2.73E-08 | 3.54E-06 |
| XR_001656640.1 | LOC107823449 | lncRNA | 102.296394 | 35.99202 | -1.50701 | 2.84E-08 | 3.66E-06 |
| TCONS_00160288 | XLOC_082320 | lncRNA | 1.398931 | 29.002415 | 4.37378 | 3.11E-08 | 3.99E-06 |
| TCONS_00021085 | XLOC_010870 | lncRNA | 37.977863 | 3.268636 | -3.5384 | 3.63E-08 | 4.64E-06 |
| TCONS_00028830 | XLOC_014862 | lncRNA | 0.452739 | 25.298851 | 5.80425 | 4.36E-08 | 5.47E-06 |
| TCONS_00101287 | XLOC_052055 | lncRNA | 0.649675 | 25.223625 | 5.27891 | 4.36E-08 | 5.47E-06 |
| TCONS_00227600 | XLOC_116452 | lncRNA | 30.603626 | 1.017462 | -4.91066 | 5.87E-08 | 7.27E-06 |
| TCONS_00159200 | XLOC_081764 | lncRNA | 1.980578 | 28.905361 | 3.86734 | 6.07E-08 | 7.49E-06 |
| TCONS_00115047 | XLOC_059025 | lncRNA | 26.167368 | 0.377735 | -6.11425 | 7.00E-08 | 8.57E-06 |
| TCONS_00137029 | XLOC_070472 | lncRNA | 26.075024 | 0.480169 | -5.76298 | 7.00E-08 | 8.57E-06 |
| TCONS_00199546 | XLOC_102176 | lncRNA | 26.249098 | 0.42108 | -5.96203 | 7.00E-08 | 8.57E-06 |
| TCONS_00020350 | XLOC_010456 | lncRNA | 0.849847 | 24.419683 | 4.8447 | 8.77E-08 | 1.06E-05 |
| TCONS_00230669 | XLOC_118019 | lncRNA | 0.940964 | 24.916029 | 4.72679 | 8.77E-08 | 1.06E-05 |
| TCONS_00077020 | XLOC_039605 | lncRNA | 0.053984 | 24.330462 | 8.81602 | 8.77E-08 | 1.06E-05 |
| TCONS_00020708 | XLOC_010669 | lncRNA | 0.691514 | 24.462576 | 5.14467 | 8.77E-08 | 1.06E-05 |
| TCONS_00157328 | XLOC_080861 | lncRNA | 0.508448 | 24.589626 | 5.59581 | 8.77E-08 | 1.06E-05 |
| TCONS_00072999 | XLOC_037468 | lncRNA | 1.91747 | 27.552559 | 3.84491 | 1.19E-07 | 1.40E-05 |
| TCONS_00135472 | XLOC_069668 | lncRNA | 25.675795 | 0.382792 | -6.0677 | 1.35E-07 | 1.57E-05 |
| TCONS_00160101 | XLOC_082191 | lncRNA | 25.297016 | 0.017931 | -10.4623 | 1.35E-07 | 1.57E-05 |
| TCONS_00209419 | XLOC_107265 | lncRNA | 25.257545 | 0.392455 | -6.00804 | 1.35E-07 | 1.57E-05 |
| TCONS_00030358 | XLOC_015617 | lncRNA | 0.783985 | 23.76536 | 4.92189 | 1.76E-07 | 2.02E-05 |
| TCONS_00120682 | XLOC_061950 | lncRNA | 0.572819 | 23.179342 | 5.33862 | 1.76E-07 | 2.02E-05 |
| TCONS_00041088 | XLOC_021178 | lncRNA | 28.666416 | 1.928744 | -3.89363 | 2.06E-07 | 2.34E-05 |
| XR_001653387.1 | LOC107809337 | lncRNA | 19.247248 | 0 | #NAME? | 2.19E-07 | 2.47E-05 |
| TCONS_00160525 | XLOC_082435 | lncRNA | 2.087897 | 29.853382 | 3.83777 | 2.21E-07 | 2.49E-05 |
| TCONS_00074624 | XLOC_038354 | lncRNA | 24.232376 | 0.399926 | -5.92106 | 2.60E-07 | 2.90E-05 |
| TCONS_00016485 | XLOC_008544 | lncRNA | 24.520166 | 0.154662 | -7.30871 | 2.60E-07 | 2.90E-05 |
| TCONS_00042423 | XLOC_021833 | lncRNA | 24.121702 | 0.504781 | -5.57853 | 2.60E-07 | 2.90E-05 |
| TCONS_00191761 | XLOC_098225 | lncRNA | 0.740336 | 22.669418 | 4.93642 | 3.54E-07 | 3.90E-05 |
| TCONS_00159386 | XLOC_081839 | lncRNA | 30.994629 | 2.203744 | -3.81399 | 4.13E-07 | 4.51E-05 |
| TCONS_00060900 | XLOC_031250 | lncRNA | 18.180843 | 0 | #NAME? | 4.38E-07 | 4.74E-05 |
| TCONS_00175661 | XLOC_089994 | lncRNA | 4.363124 | 33.09016 | 2.92297 | 4.47E-07 | 4.82E-05 |
| TCONS_00116965 | XLOC_060020 | lncRNA | 23.242907 | 0.406867 | -5.83609 | 4.99E-07 | 5.34E-05 |
| TCONS_00137769 | XLOC_070858 | lncRNA | 23.369389 | 0.385227 | -5.92277 | 4.99E-07 | 5.34E-05 |
| TCONS_00112329 | XLOC_057629 | lncRNA | 23.350485 | 0.873174 | -4.74104 | 4.99E-07 | 5.34E-05 |
| TCONS_00116194 | XLOC_059615 | lncRNA | 5.234342 | 35.699696 | 2.76983 | 5.42E-07 | 5.77E-05 |
| XR_001650896.1 | LOC107798479 | lncRNA | 42.490181 | 7.102255 | -2.58078 | 6.09E-07 | 6.47E-05 |
| TCONS_00042732 | XLOC_021991 | lncRNA | 0.024604 | 21.973404 | 9.80265 | 7.10E-07 | 7.45E-05 |
| TCONS_00203853 | XLOC_104423 | lncRNA | 0.204871 | 21.643581 | 6.72308 | 7.10E-07 | 7.45E-05 |
| TCONS_00129360 | XLOC_066673 | lncRNA | 22.489246 | 0.602385 | -5.22241 | 9.60E-07 | 9.73E-05 |
| TCONS_00067334 | XLOC_034575 | lncRNA | 25.630386 | 1.700858 | -3.91352 | 1.34E-06 | 0.000132711 |
| TCONS_00154911 | XLOC_079581 | lncRNA | 0.838772 | 20.266989 | 4.59471 | 1.42E-06 | 0.000140318 |
| TCONS_00031396 | XLOC_016143 | lncRNA | 21.24835 | 0.567624 | -5.22627 | 1.84E-06 | 0.000177387 |
| XR_001651265.1 | LOC107800022 | lncRNA | 6.599072 | 35.932224 | 2.44494 | 1.90E-06 | 0.000182247 |
| TCONS_00154467 | XLOC_079348 | lncRNA | 8.192863 | 39.376293 | 2.26489 | 2.03E-06 | 0.000193981 |
| TCONS_00193578 | XLOC_099123 | lncRNA | 27.442286 | 2.180827 | -3.65345 | 2.48E-06 | 0.000235689 |
| XR_001647555.1 | LOC107783870 | lncRNA | 2.648581 | 25.737854 | 3.2806 | 2.82E-06 | 0.00026721 |
| TCONS_00045168 | XLOC_023199 | lncRNA | 0.057094 | 19.153292 | 8.39004 | 2.85E-06 | 0.000267537 |
| TCONS_00224943 | XLOC_115144 | lncRNA | 0.821675 | 19.10387 | 4.53915 | 2.85E-06 | 0.000267537 |
| TCONS_00174795 | XLOC_089573 | lncRNA | 0.894076 | 19.481213 | 4.44554 | 2.85E-06 | 0.000267537 |
| TCONS_00124928 | XLOC_064262 | lncRNA | 1.101428 | 22.657423 | 4.36254 | 3.27E-06 | 0.000304646 |
| TCONS_00015114 | XLOC_007803 | lncRNA | 0 | 14.427065 | inf | 3.62E-06 | 0.000328064 |
| XR_001653523.1 | LOC107809951 | lncRNA | 0.478276 | 18.016363 | 5.23532 | 5.71E-06 | 0.00050379 |
| TCONS_00186403 | XLOC_095562 | lncRNA | 0.49239 | 18.150312 | 5.20405 | 5.71E-06 | 0.00050379 |
| TCONS_00034851 | XLOC_017941 | lncRNA | 0.954183 | 18.227442 | 4.2557 | 5.71E-06 | 0.00050379 |
| XR_001642604.1 | LOC107761477 | lncRNA | 33.391052 | 5.681575 | -2.5551 | 6.19E-06 | 0.000544894 |
| TCONS_00155525 | XLOC_079900 | lncRNA | 28.254715 | 3.462999 | -3.0284 | 6.64E-06 | 0.00058165 |
| TCONS_00107058 | XLOC_054924 | lncRNA | 19.8904 | 0.487242 | -5.35129 | 6.79E-06 | 0.000590166 |
| TCONS_00211541 | XLOC_108286 | lncRNA | 19.096992 | 0.830422 | -4.52336 | 6.79E-06 | 0.000590166 |
| TCONS_00131187 | XLOC_067557 | lncRNA | 19.489828 | 0.523152 | -5.21935 | 6.79E-06 | 0.000590166 |
| XR_001658616.1 | LOC107832645 | lncRNA | 14.403399 | 0 | #NAME? | 7.18E-06 | 0.000616759 |
| TCONS_00231049 | XLOC_118186 | lncRNA | 25.414009 | 2.759219 | -3.20329 | 8.08E-06 | 0.000683853 |
| TCONS_00171235 | XLOC_087792 | lncRNA | 30.228189 | 4.177778 | -2.85509 | 8.71E-06 | 0.000731969 |
| XR_001651555.1 | LOC107801115 | lncRNA | 2.360737 | 23.29158 | 3.3025 | 9.93E-06 | 0.000826786 |
| TCONS_00201106 | XLOC_103007 | lncRNA | 2.684423 | 23.777464 | 3.14691 | 9.93E-06 | 0.000826786 |
| XR_001649948.1 | LOC107794550 | lncRNA | 6.835734 | 32.288132 | 2.23984 | 1.00E-05 | 0.00083197 |
| TCONS_00052222 | XLOC_026772 | lncRNA | 0.517736 | 17.715046 | 5.09661 | 1.14E-05 | 0.000940073 |
| TCONS_00202779 | XLOC_103868 | lncRNA | 1.591982 | 20.71629 | 3.70187 | 1.22E-05 | 0.000998665 |
| TCONS_00103275 | XLOC_053119 | lncRNA | 18.987303 | 0.665249 | -4.835 | 1.30E-05 | 0.001059062 |
| TCONS_00135470 | XLOC_069669 | lncRNA | 18.634987 | 0.946692 | -4.29897 | 1.30E-05 | 0.001059062 |
| TCONS_00193497 | XLOC_099081 | lncRNA | 18.666517 | 0.576336 | -5.0174 | 1.30E-05 | 0.001059062 |
| TCONS_00216729 | XLOC_110902 | lncRNA | 18.429874 | 0.691004 | -4.73721 | 1.30E-05 | 0.001059062 |
| TCONS_00075250 | XLOC_038678 | lncRNA | 0.576923 | 16.118868 | 4.80423 | 2.28E-05 | 0.001783908 |
| TCONS_00087771 | XLOC_044991 | lncRNA | 0.703044 | 16.589628 | 4.56052 | 2.28E-05 | 0.001783908 |
| TCONS_00042912 | XLOC_022090 | lncRNA | 0.718182 | 16.341593 | 4.50806 | 2.28E-05 | 0.001783908 |
| XR_001657493.1 | LOC107827297 | lncRNA | 3.909556 | 24.251362 | 2.63299 | 2.37E-05 | 0.001845597 |
| TCONS_00211770 | XLOC_108419 | lncRNA | 17.98646 | 0.56296 | -4.99774 | 2.49E-05 | 0.001931865 |
| TCONS_00027308 | XLOC_014075 | lncRNA | 3.740261 | 23.750496 | 2.66675 | 4.29E-05 | 0.003171366 |
| TCONS_00206157 | XLOC_105598 | lncRNA | 27.37236 | 4.096277 | -2.74033 | 4.41E-05 | 0.003256012 |
| TCONS_00140197 | XLOC_072139 | lncRNA | 1.565461 | 18.965836 | 3.59874 | 4.48E-05 | 0.003282744 |
| TCONS_00031682 | XLOC_016309 | lncRNA | 0.47246 | 15.699932 | 5.05442 | 4.56E-05 | 0.003305001 |
| TCONS_00240154 | XLOC_122848 | lncRNA | 0.689774 | 15.243702 | 4.46595 | 4.56E-05 | 0.003305001 |
| TCONS_00155101 | XLOC_079683 | lncRNA | 0.748168 | 15.279881 | 4.35213 | 4.56E-05 | 0.003305001 |
| TCONS_00002994 | XLOC_001556 | lncRNA | 0.511891 | 15.626281 | 4.93199 | 4.56E-05 | 0.003305001 |
| TCONS_00166049 | XLOC_085214 | lncRNA | 22.704559 | 2.017606 | -3.49227 | 4.65E-05 | 0.003356712 |
| TCONS_00067349 | XLOC_034586 | lncRNA | 16.13574 | 0.249252 | -6.01651 | 4.77E-05 | 0.003409639 |
| TCONS_00164843 | XLOC_084617 | lncRNA | 2.239302 | 20.751516 | 3.2121 | 6.38E-05 | 0.004436085 |
| TCONS_00215207 | XLOC_110162 | lncRNA | 21.277227 | 2.260566 | -3.23455 | 8.29E-05 | 0.005592342 |
| TCONS_00150820 | XLOC_077501 | lncRNA | 21.545086 | 2.919058 | -2.88378 | 8.29E-05 | 0.005592342 |
| XR_001643946.1 | LOC107767584 | lncRNA | 1.546696 | 17.316946 | 3.48492 | 8.55E-05 | 0.005736298 |
| TCONS_00100881 | XLOC_051857 | lncRNA | 1.252098 | 17.124094 | 3.77361 | 8.55E-05 | 0.005736298 |
| TCONS_00071376 | XLOC_036626 | lncRNA | 0.477239 | 14.740682 | 4.94895 | 9.08E-05 | 0.005960213 |
| TCONS_00136711 | XLOC_070297 | lncRNA | 0.836487 | 14.888906 | 4.15375 | 9.08E-05 | 0.005960213 |
| TCONS_00195379 | XLOC_100026 | lncRNA | 0.701374 | 14.329985 | 4.35271 | 9.08E-05 | 0.005960213 |
| TCONS_00187874 | XLOC_096267 | lncRNA | 0.251576 | 14.785548 | 5.87705 | 9.08E-05 | 0.005960213 |
| TCONS_00042218 | XLOC_021732 | lncRNA | 0.117634 | 14.573068 | 6.95286 | 9.08E-05 | 0.005960213 |
| TCONS_00151960 | XLOC_078062 | lncRNA | 15.16551 | 0.378762 | -5.32336 | 9.11E-05 | 0.005960213 |
| TCONS_00090106 | XLOC_046203 | lncRNA | 15.135037 | 0.421458 | -5.16636 | 9.11E-05 | 0.005960213 |
| TCONS_00095522 | XLOC_049007 | lncRNA | 15.226 | 0.758181 | -4.32785 | 9.11E-05 | 0.005960213 |
| TCONS_00129324 | XLOC_066655 | lncRNA | 15.961184 | 0.396977 | -5.32937 | 9.11E-05 | 0.005960213 |
| TCONS_00030826 | XLOC_015843 | lncRNA | 15.374743 | 0.389127 | -5.30418 | 9.11E-05 | 0.005960213 |
| TCONS_00199456 | XLOC_102128 | lncRNA | 14.463396 | 0.776273 | -4.2197 | 0.000173837 | 0.010703493 |
| TCONS_00205844 | XLOC_105414 | lncRNA | 14.742215 | 0.47919 | -4.94321 | 0.000173837 | 0.010703493 |
| TCONS_00233635 | XLOC_119507 | lncRNA | 14.896797 | 0.627086 | -4.5702 | 0.000173837 | 0.010703493 |
| TCONS_00108674 | XLOC_055744 | lncRNA | 14.757891 | 0.381399 | -5.27404 | 0.000173837 | 0.010703493 |
| XR_001645621.1 | LOC107774976 | lncRNA | 47.751003 | 16.721828 | -1.5138 | 0.000175342 | 0.010785702 |
| TCONS_00197588 | XLOC_101167 | lncRNA | 17.617517 | 1.383828 | -3.67027 | 0.0001797 | 0.011006379 |
| TCONS_00164902 | XLOC_084658 | lncRNA | 0.864482 | 13.023932 | 3.91319 | 0.000180653 | 0.011006379 |
| TCONS_00103329 | XLOC_053142 | lncRNA | 0.171084 | 13.035392 | 6.25159 | 0.000180653 | 0.011006379 |
| XR_001657220.1 | LOC107826167 | lncRNA | 9.008235 | 0 | #NAME? | 0.000247591 | 0.014590552 |
| TCONS_00127506 | XLOC_065726 | lncRNA | 9.970854 | 0 | #NAME? | 0.000247591 | 0.014590552 |
| TCONS_00088283 | XLOC_045282 | lncRNA | 3.370797 | 20.875677 | 2.63066 | 0.000247708 | 0.014590552 |
| TCONS_00201999 | XLOC_103470 | lncRNA | 5.087125 | 24.323395 | 2.25742 | 0.000257222 | 0.015064381 |
| TCONS_00033708 | XLOC_017378 | lncRNA | 1.249977 | 15.116959 | 3.59619 | 0.000308786 | 0.017746176 |
| TCONS_00066923 | XLOC_034357 | lncRNA | 1.171481 | 15.39326 | 3.71589 | 0.000308786 | 0.017746176 |
| TCONS_00125217 | XLOC_064425 | lncRNA | 16.113031 | 1.322024 | -3.60741 | 0.000327579 | 0.018671069 |
| TCONS_00193581 | XLOC_099125 | lncRNA | 13.823302 | 0.467748 | -4.88523 | 0.000331217 | 0.018671069 |
| TCONS_00091893 | XLOC_047118 | lncRNA | 13.748228 | 0.382423 | -5.16793 | 0.000331217 | 0.018671069 |
| TCONS_00123742 | XLOC_063545 | lncRNA | 13.639744 | 0.760924 | -4.16392 | 0.000331217 | 0.018671069 |
| TCONS_00197652 | XLOC_101213 | lncRNA | 13.133319 | 0.735196 | -4.15896 | 0.000331217 | 0.018671069 |
| TCONS_00137257 | XLOC_070571 | lncRNA | 13.557261 | 0.442764 | -4.93638 | 0.000331217 | 0.018671069 |
| TCONS_00159775 | XLOC_082036 | lncRNA | 13.691216 | 0.913522 | -3.90567 | 0.000331217 | 0.018671069 |
| XR_001654396.1 | LOC107813625 | lncRNA | 0 | 8.228928 | inf | 0.000333067 | 0.018671069 |
| TCONS_00190849 | XLOC_097737 | lncRNA | 0.550046 | 12.388579 | 4.49331 | 0.000358916 | 0.019789966 |
| TCONS_00194764 | XLOC_099691 | lncRNA | 0.473039 | 12.590586 | 4.73424 | 0.000358916 | 0.019789966 |
| TCONS_00106660 | XLOC_054740 | lncRNA | 0.521547 | 12.386871 | 4.56987 | 0.000358916 | 0.019789966 |
| TCONS_00047460 | XLOC_024358 | lncRNA | 0.589229 | 12.43628 | 4.39958 | 0.000358916 | 0.019789966 |
| TCONS_00002285 | XLOC_001187 | lncRNA | 0.604878 | 12.249681 | 4.33996 | 0.000358916 | 0.019789966 |
| TCONS_00196791 | XLOC_100742 | lncRNA | 0.518942 | 12.144588 | 4.5486 | 0.000358916 | 0.019789966 |
| TCONS_00123487 | XLOC_063390 | lncRNA | 3.533216 | 19.266804 | 2.44706 | 0.000439697 | 0.023934062 |
| TCONS_00035413 | XLOC_018235 | lncRNA | 3.286871 | 19.172703 | 2.54427 | 0.000439697 | 0.023934062 |
| TCONS_00195751 | XLOC_100225 | lncRNA | 8.499628 | 0 | #NAME? | 0.000507669 | 0.026944876 |
| TCONS_00087782 | XLOC_044998 | lncRNA | 20.387625 | 3.705448 | -2.45997 | 0.000548761 | 0.029053371 |
| TCONS_00028996 | XLOC_014936 | lncRNA | 30.584066 | 8.583052 | -1.83322 | 0.000574916 | 0.030312471 |
| XR_001651764.1 | LOC107802068 | lncRNA | 1.056171 | 14.974597 | 3.8256 | 0.000583274 | 0.030551316 |
| TCONS_00089973 | XLOC_046130 | lncRNA | 1.038748 | 14.332043 | 3.78633 | 0.000583274 | 0.030551316 |
| TCONS_00063969 | XLOC_032792 | lncRNA | 1.796087 | 14.073311 | 2.97003 | 0.000583274 | 0.030551316 |
| TCONS_00190614 | XLOC_097626 | lncRNA | 1.591416 | 14.641025 | 3.20163 | 0.000583274 | 0.030551316 |
| TCONS_00022761 | XLOC_011718 | lncRNA | 1.023713 | 14.043253 | 3.77799 | 0.000583274 | 0.030551316 |
| TCONS_00102131 | XLOC_052523 | lncRNA | 15.377809 | 1.714392 | -3.16508 | 0.000594994 | 0.03108869 |
| XR_001647406.1 | LOC107783192 | lncRNA | 12.526264 | 0.213816 | -5.87244 | 0.00063006 | 0.032338698 |
| TCONS_00196032 | XLOC_100378 | lncRNA | 12.721061 | 0.593067 | -4.42288 | 0.00063006 | 0.032338698 |
| TCONS_00060822 | XLOC_031217 | lncRNA | 12.304134 | 0.447883 | -4.77988 | 0.00063006 | 0.032338698 |
| TCONS_00048353 | XLOC_024807 | lncRNA | 12.458511 | 0.431343 | -4.85215 | 0.00063006 | 0.032338698 |
| TCONS_00115309 | XLOC_059158 | lncRNA | 12.108521 | 0.142489 | -6.40903 | 0.00063006 | 0.032338698 |
| TCONS_00028871 | XLOC_014878 | lncRNA | 12.360429 | 0.617623 | -4.32286 | 0.00063006 | 0.032338698 |
| TCONS_00089727 | XLOC_045996 | lncRNA | 12.430959 | 0.520499 | -4.5779 | 0.00063006 | 0.032338698 |
| TCONS_00185856 | XLOC_095270 | lncRNA | 12.049948 | 0.799776 | -3.91329 | 0.00063006 | 0.032338698 |
| XR_001657317.1 | LOC107826636 | lncRNA | 0.522371 | 11.387224 | 4.4462 | 0.000711763 | 0.035581817 |
| TCONS_00051801 | XLOC_026552 | lncRNA | 0.732559 | 11.443987 | 3.9655 | 0.000711763 | 0.035581817 |
| TCONS_00196005 | XLOC_100365 | lncRNA | 0.83388 | 11.127223 | 3.73811 | 0.000711763 | 0.035581817 |
| TCONS_00059988 | XLOC_030777 | lncRNA | 0.581494 | 11.529792 | 4.30946 | 0.000711763 | 0.035581817 |
| TCONS_00074636 | XLOC_038362 | lncRNA | 0.434304 | 11.896561 | 4.7757 | 0.000711763 | 0.035581817 |
| TCONS_00087249 | XLOC_044716 | lncRNA | 0.729841 | 11.84854 | 4.02098 | 0.000711763 | 0.035581817 |
| TCONS_00094375 | XLOC_048396 | lncRNA | 0.63101 | 11.629815 | 4.20402 | 0.000711763 | 0.035581817 |
| TCONS_00120959 | XLOC_062081 | lncRNA | 0.633616 | 11.474098 | 4.17863 | 0.000711763 | 0.035581817 |
| XR_001645115.1 | LOC107772613 | lncRNA | 0 | 7.010348 | inf | 0.000717209 | 0.035581817 |
| TCONS_00123631 | XLOC_063475 | lncRNA | 0 | 7.923275 | inf | 0.000717209 | 0.035581817 |
| TCONS_00118840 | XLOC_060999 | lncRNA | 0 | 7.815729 | inf | 0.000717209 | 0.035581817 |
| TCONS_00199444 | XLOC_102125 | lncRNA | 0 | 7.383526 | inf | 0.000717209 | 0.035581817 |
| TCONS_00072189 | XLOC_037043 | lncRNA | 19.69919 | 3.38988 | -2.53883 | 0.000933139 | 0.045343162 |
| TCONS_00043988 | XLOC_022597 | lncRNA | 19.320568 | 3.057186 | -2.65986 | 0.000933139 | 0.045343162 |
| TCONS_00151115 | XLOC_077665 | lncRNA | 19.299299 | 3.55078 | -2.44234 | 0.000933139 | 0.045343162 |
| TCONS_00118287 | XLOC_060702 | lncRNA | 7.041655 | 0 | #NAME? | 0.001046038 | 0.049917431 |
