# Supplementary material for: Genome-wide identification and functional prediction of tobacco lncRNAs responsive to root-knot nematode stress
Source: PLoS One. 2018 Nov 14;13(11):e0204506. doi: 10.1371/journal.pone.0204506 (PMC6235259; doi:10.1371/journal.pone.0204506)
Supplement: S3 Table — (DOC) [file pone.0204506.s005.doc]

**S3 Table. Significantly differentially expressed lncRNAs in Long bohuang.**

| Transcript | Gene | Type | longCK | longRKN | log2(foldchange) | P-value | Q-value |
| --- | --- | --- | --- | --- | --- | --- | --- |
| XR_001657700.1 | LOC107828270 | lncRNA | 0 | 441.631714 | inf | 0 | 0 |
| XR_001643599.1 | LOC107766141 | lncRNA | 16.521894 | 183.719849 | 3.47506 | 0 | 0 |
| XR_001653261.1 | LOC107808800 | lncRNA | 607.295776 | 1716.583008 | 1.49907 | 0 | 0 |
| XR_001648910.1 | LOC107789767 | lncRNA | 4.369175 | 105.609047 | 4.59523 | 0 | 0 |
| XR_001658459.1 | LOC107831913 | lncRNA | 3719.412109 | 12974.43066 | 1.80252 | 0 | 0 |
| XR_001645212.1 | LOC107773004 | lncRNA | 245.991516 | 47.736454 | -2.36545 | 0 | 0 |
| TCONS_00120663 | XLOC_061941 | lncRNA | 1626.743164 | 3.445857 | -8.88291 | 0 | 0 |
| TCONS_00171247 | XLOC_087795 | lncRNA | 1280.793457 | 62.952744 | -4.34662 | 0 | 0 |
| TCONS_00171249 | XLOC_087798 | lncRNA | 3.922292 | 2453.724609 | 9.28906 | 0 | 0 |
| TCONS_00171248 | XLOC_087795 | lncRNA | 0.148011 | 1304.133545 | 13.1051 | 0 | 0 |
| TCONS_00011888 | XLOC_006162 | lncRNA | 97.063179 | 0.314379 | -8.27028 | 0 | 0 |
| TCONS_00239960 | XLOC_122757 | lncRNA | 0.407362 | 56.450375 | 7.11453 | 0 | 0 |
| TCONS_00099248 | XLOC_050970 | lncRNA | 5363.24707 | 0.474914 | -13.4632 | 0 | 0 |
| TCONS_00204550 | XLOC_104785 | lncRNA | 209.700531 | 0.337979 | -9.27718 | 0 | 0 |
| TCONS_00190599 | XLOC_097619 | lncRNA | 7.522082 | 162.967285 | 4.43731 | 0 | 0 |
| TCONS_00197582 | XLOC_101167 | lncRNA | 107.875298 | 1.853659 | -5.86284 | 0 | 0 |
| TCONS_00135472 | XLOC_069668 | lncRNA | 74.280121 | 0.787327 | -6.55987 | 0 | 0 |
| TCONS_00000853 | XLOC_000473 | lncRNA | 0.501982 | 223.754593 | 8.80007 | 0 | 0 |
| TCONS_00066901 | XLOC_034346 | lncRNA | 98.194809 | 0.619891 | -7.30749 | 0 | 0 |
| TCONS_00031681 | XLOC_016308 | lncRNA | 172.634064 | 0.644744 | -8.06478 | 0 | 0 |
| TCONS_00080458 | XLOC_041265 | lncRNA | 0.964495 | 2735.350586 | 11.4697 | 0 | 0 |
| TCONS_00009042 | XLOC_004703 | lncRNA | 84.329567 | 0.269905 | -8.28744 | 0 | 0 |
| TCONS_00042728 | XLOC_021989 | lncRNA | 81.408226 | 0.265475 | -8.26045 | 0 | 0 |
| TCONS_00031624 | XLOC_016278 | lncRNA | 0.733251 | 4105.598145 | 12.451 | 0 | 0 |
| TCONS_00031623 | XLOC_016277 | lncRNA | 2.034956 | 6125.434082 | 11.5556 | 0 | 0 |
| TCONS_00132003 | XLOC_067967 | lncRNA | 3498.796143 | 0.579202 | -12.5605 | 0 | 0 |
| TCONS_00132006 | XLOC_067969 | lncRNA | 10.659205 | 8122.958984 | 9.57376 | 0 | 0 |
| TCONS_00061324 | XLOC_031466 | lncRNA | 0.359335 | 335.191986 | 9.86544 | 0 | 0 |
| TCONS_00115994 | XLOC_059513 | lncRNA | 0.420959 | 100.09211 | 7.89343 | 0 | 0 |
| TCONS_00240165 | XLOC_122856 | lncRNA | 2022.085693 | 1.972861 | -10.0013 | 0 | 0 |
| TCONS_00083548 | XLOC_042861 | lncRNA | 458.69812 | 0.284502 | -10.6549 | 0 | 0 |
| TCONS_00208482 | XLOC_106773 | lncRNA | 1.208571 | 599.35376 | 8.95396 | 0 | 0 |
| TCONS_00028139 | XLOC_014484 | lncRNA | 2.682222 | 797.879456 | 8.2166 | 0 | 0 |
| TCONS_00145586 | XLOC_074794 | lncRNA | 72.034798 | 0.459608 | -7.29215 | 0 | 0 |
| TCONS_00031030 | XLOC_015950 | lncRNA | 0.576967 | 56.323753 | 6.60911 | 0 | 0 |
| TCONS_00017656 | XLOC_009146 | lncRNA | 133.325348 | 0.305445 | -8.76982 | 0 | 0 |
| TCONS_00205845 | XLOC_105415 | lncRNA | 1621.019287 | 0.292473 | -12.4363 | 0 | 0 |
| TCONS_00205846 | XLOC_105414 | lncRNA | 120.467842 | 0.513271 | -7.87471 | 0 | 0 |
| TCONS_00205843 | XLOC_105414 | lncRNA | 4.473868 | 237.917404 | 5.73279 | 0 | 0 |
| TCONS_00164902 | XLOC_084658 | lncRNA | 3.775246 | 3020.700439 | 9.6441 | 0 | 0 |
| TCONS_00147604 | XLOC_075834 | lncRNA | 84.015297 | 1.371211 | -5.93713 | 0 | 0 |
| TCONS_00074609 | XLOC_038353 | lncRNA | 0.368032 | 415.469513 | 10.1407 | 0 | 0 |
| TCONS_00041708 | XLOC_021488 | lncRNA | 0.43672 | 760.207092 | 10.7655 | 0 | 0 |
| TCONS_00092024 | XLOC_047184 | lncRNA | 0.407528 | 202.458572 | 8.95651 | 0 | 0 |
| TCONS_00120712 | XLOC_061964 | lncRNA | 146.516098 | 0.297247 | -8.94518 | 0 | 0 |
| TCONS_00124928 | XLOC_064262 | lncRNA | 91.904076 | 0.339087 | -8.08233 | 0 | 0 |
| TCONS_00168067 | XLOC_086279 | lncRNA | 0.427917 | 185.629745 | 8.76088 | 0 | 0 |
| TCONS_00074220 | XLOC_038141 | lncRNA | 0.960097 | 56.279202 | 5.87328 | 0 | 0 |
| TCONS_00048140 | XLOC_024715 | lncRNA | 1.118555 | 130.065201 | 6.86146 | 0 | 0 |
| TCONS_00035882 | XLOC_018489 | lncRNA | 196.916534 | 0.433745 | -8.82652 | 0 | 0 |
| TCONS_00025359 | XLOC_013097 | lncRNA | 0.394663 | 54.129635 | 7.09965 | 0 | 0 |
| TCONS_00157333 | XLOC_080861 | lncRNA | 7.75922 | 110.637299 | 3.83378 | 0 | 0 |
| TCONS_00130884 | XLOC_067417 | lncRNA | 152.756241 | 0.288983 | -9.04603 | 0 | 0 |
| TCONS_00017468 | XLOC_009063 | lncRNA | 0.830225 | 82.69352 | 6.63813 | 0 | 0 |
| TCONS_00223690 | XLOC_114481 | lncRNA | 0 | 661.598694 | inf | 0 | 0 |
| TCONS_00032608 | XLOC_016809 | lncRNA | 0.372368 | 267.443207 | 9.48829 | 0 | 0 |
| TCONS_00120519 | XLOC_061860 | lncRNA | 7.151366 | 3588.614502 | 8.97099 | 0 | 0 |
| TCONS_00186947 | XLOC_095821 | lncRNA | 163.446213 | 0.51699 | -8.30446 | 0 | 0 |
| TCONS_00125091 | XLOC_064357 | lncRNA | 0.524232 | 455.204803 | 9.76209 | 0 | 0 |
| TCONS_00161856 | XLOC_083107 | lncRNA | 5.521786 | 227.60994 | 5.36528 | 0 | 0 |
| TCONS_00161858 | XLOC_083109 | lncRNA | 0.364705 | 6434.196777 | 14.1067 | 0 | 0 |
| TCONS_00065541 | XLOC_033612 | lncRNA | 4.453156 | 111.140472 | 4.64141 | 0 | 0 |
| TCONS_00000797 | XLOC_000435 | lncRNA | 379.612061 | 1.813658 | -7.70948 | 0 | 0 |
| TCONS_00095157 | XLOC_048813 | lncRNA | 0.371473 | 341.957489 | 9.84634 | 0 | 0 |
| TCONS_00110406 | XLOC_056626 | lncRNA | 86.49617 | 0 | #NAME? | 0 | 0 |
| TCONS_00085598 | XLOC_043908 | lncRNA | 1.29892 | 704.931946 | 9.08403 | 0 | 0 |
| TCONS_00025335 | XLOC_013082 | lncRNA | 0.413152 | 218.884445 | 9.04928 | 0 | 0 |
| TCONS_00025334 | XLOC_013081 | lncRNA | 0.438927 | 4542.306641 | 13.3372 | 0 | 0 |
| TCONS_00025330 | XLOC_013083 | lncRNA | 0.528642 | 72.678749 | 7.1031 | 0 | 0 |
| TCONS_00125089 | XLOC_064356 | lncRNA | 0.639854 | 12689.11816 | 14.2755 | 0 | 0 |
| TCONS_00161846 | XLOC_083109 | lncRNA | 3036.049072 | 4.646647 | -9.35179 | 0 | 0 |
| TCONS_00005788 | XLOC_003006 | lncRNA | 0.181124 | 387.321442 | 11.0623 | 0 | 0 |
| TCONS_00015154 | XLOC_007828 | lncRNA | 603.306641 | 23.085772 | -4.70782 | 0 | 0 |
| TCONS_00130734 | XLOC_067356 | lncRNA | 1.346764 | 532.48468 | 8.6271 | 0 | 0 |
| TCONS_00216972 | XLOC_111009 | lncRNA | 193.888275 | 0.272009 | -9.47736 | 0 | 0 |
| TCONS_00227190 | XLOC_116259 | lncRNA | 100.10952 | 0.284973 | -8.45654 | 0 | 0 |
| TCONS_00198762 | XLOC_101777 | lncRNA | 12678.66211 | 0.647827 | -14.2564 | 0 | 0 |
| TCONS_00059683 | XLOC_030604 | lncRNA | 0.588563 | 85.107399 | 7.17594 | 0 | 0 |
| TCONS_00152172 | XLOC_078169 | lncRNA | 1022.40448 | 0.315674 | -11.6612 | 0 | 0 |
| TCONS_00190901 | XLOC_097769 | lncRNA | 0.508124 | 63.243053 | 6.95958 | 0 | 0 |
| TCONS_00102490 | XLOC_052697 | lncRNA | 1.047342 | 62.482193 | 5.89864 | 0 | 0 |
| TCONS_00054097 | XLOC_027683 | lncRNA | 836.280273 | 0.554105 | -10.5596 | 0 | 0 |
| TCONS_00054094 | XLOC_027682 | lncRNA | 1074.54126 | 0.917553 | -10.1936 | 0 | 0 |
| TCONS_00054099 | XLOC_027683 | lncRNA | 0.491444 | 1669.027222 | 11.7297 | 0 | 0 |
| TCONS_00010582 | XLOC_005508 | lncRNA | 94.088799 | 0.306781 | -8.26067 | 0 | 0 |
| TCONS_00048989 | XLOC_025136 | lncRNA | 84.285095 | 1.738286 | -5.59954 | 0 | 0 |
| TCONS_00000826 | XLOC_000434 | lncRNA | 171.191605 | 1.210675 | -7.14366 | 0 | 0 |
| TCONS_00017239 | XLOC_008938 | lncRNA | 0.723668 | 619.570068 | 9.74172 | 0 | 0 |
| TCONS_00233714 | XLOC_119538 | lncRNA | 37887.48047 | 0.56646 | -16.0294 | 0 | 0 |
| TCONS_00199181 | XLOC_102013 | lncRNA | 0.352654 | 634.494263 | 10.8131 | 0 | 0 |
| TCONS_00078284 | XLOC_040206 | lncRNA | 0.386446 | 103.542801 | 8.06574 | 0 | 0 |
| TCONS_00065542 | XLOC_033613 | lncRNA | 0.74192 | 9681.489258 | 13.6717 | 0 | 0 |
| TCONS_00220354 | XLOC_112767 | lncRNA | 3.101024 | 74.429169 | 4.58505 | 0 | 0 |
| TCONS_00166396 | XLOC_085392 | lncRNA | 93.775665 | 0.745278 | -6.97529 | 0 | 0 |
| TCONS_00110525 | XLOC_056665 | lncRNA | 0.223127 | 60.486362 | 8.0826 | 0 | 0 |
| TCONS_00031688 | XLOC_016311 | lncRNA | 0.428925 | 283.341888 | 9.3676 | 0 | 0 |
| TCONS_00243837 | XLOC_124742 | lncRNA | 117.907951 | 0.141143 | -9.70629 | 0 | 0 |
| TCONS_00091328 | XLOC_046842 | lncRNA | 62.931538 | 0.260695 | -7.91528 | 1.11E-16 | 3.12E-14 |
| TCONS_00140692 | XLOC_072371 | lncRNA | 0.401045 | 47.927307 | 6.90094 | 3.33E-16 | 9.23E-14 |
| TCONS_00152788 | XLOC_078469 | lncRNA | 0 | 41.038166 | inf | 4.44E-16 | 1.19E-13 |
| TCONS_00020338 | XLOC_010459 | lncRNA | 0 | 40.73407 | inf | 1.11E-15 | 2.88E-13 |
| TCONS_00224868 | XLOC_115114 | lncRNA | 0.789199 | 44.529621 | 5.81823 | 3.11E-15 | 7.81E-13 |
| TCONS_00060900 | XLOC_031250 | lncRNA | 48.735023 | 0 | #NAME? | 1.09E-14 | 2.63E-12 |
| TCONS_00154911 | XLOC_079581 | lncRNA | 64.119553 | 2.360535 | -4.76358 | 1.31E-14 | 3.13E-12 |
| TCONS_00104117 | XLOC_053504 | lncRNA | 71.685066 | 4.740222 | -3.91865 | 1.92E-14 | 4.54E-12 |
| TCONS_00112913 | XLOC_057941 | lncRNA | 0.826237 | 41.218678 | 5.6406 | 3.20E-14 | 7.40E-12 |
| TCONS_00060978 | XLOC_031296 | lncRNA | 58.472191 | 1.750237 | -5.06213 | 3.45E-14 | 7.96E-12 |
| TCONS_00243835 | XLOC_124741 | lncRNA | 52.813046 | 0.430108 | -6.94005 | 6.47E-14 | 1.45E-11 |
| TCONS_00191543 | XLOC_098111 | lncRNA | 51.825066 | 0.998581 | -5.69763 | 1.18E-13 | 2.60E-11 |
| TCONS_00103329 | XLOC_053142 | lncRNA | 70.830063 | 5.174751 | -3.7748 | 2.19E-13 | 4.75E-11 |
| TCONS_00179486 | XLOC_092022 | lncRNA | 0.386454 | 38.916656 | 6.65395 | 3.26E-13 | 6.94E-11 |
| TCONS_00217557 | XLOC_111327 | lncRNA | 49.855968 | 0.264879 | -7.55629 | 3.92E-13 | 8.25E-11 |
| XR_001654635.1 | LOC107814774 | lncRNA | 123.651268 | 26.471731 | -2.22375 | 6.42E-13 | 1.34E-10 |
| XR_001643189.1 | LOC107764149 | lncRNA | 3.418293 | 45.253075 | 3.72667 | 2.36E-12 | 4.76E-10 |
| TCONS_00210732 | XLOC_107917 | lncRNA | 0 | 30.159561 | inf | 3.09E-12 | 6.18E-10 |
| TCONS_00200850 | XLOC_102858 | lncRNA | 0.571806 | 35.708218 | 5.96459 | 3.30E-12 | 6.59E-10 |
| XR_001654270.1 | LOC107813192 | lncRNA | 64.189537 | 5.754014 | -3.4797 | 5.43E-12 | 1.07E-09 |
| TCONS_00149348 | XLOC_076723 | lncRNA | 44.402428 | 0.617636 | -6.16774 | 7.89E-12 | 1.52E-09 |
| TCONS_00095573 | XLOC_049029 | lncRNA | 44.356159 | 0.491978 | -6.4944 | 7.89E-12 | 1.52E-09 |
| TCONS_00034610 | XLOC_017810 | lncRNA | 0.92695 | 33.73632 | 5.18567 | 1.55E-11 | 2.92E-09 |
| TCONS_00092978 | XLOC_047660 | lncRNA | 0.471417 | 33.233677 | 6.1395 | 1.55E-11 | 2.92E-09 |
| TCONS_00172114 | XLOC_088246 | lncRNA | 1.403015 | 36.485298 | 4.70071 | 2.29E-11 | 4.22E-09 |
| TCONS_00231140 | XLOC_118228 | lncRNA | 42.61808 | 0.535969 | -6.31317 | 2.62E-11 | 4.74E-09 |
| TCONS_00105574 | XLOC_054226 | lncRNA | 42.721733 | 0.337822 | -6.98256 | 2.62E-11 | 4.74E-09 |
| TCONS_00106633 | XLOC_054729 | lncRNA | 42.267368 | 0.369706 | -6.83702 | 2.62E-11 | 4.74E-09 |
| TCONS_00014809 | XLOC_007656 | lncRNA | 42.143253 | 0.735989 | -5.83947 | 2.62E-11 | 4.74E-09 |
| TCONS_00087771 | XLOC_044991 | lncRNA | 0.0731 | 32.725643 | 8.80633 | 3.34E-11 | 6.03E-09 |
| TCONS_00075665 | XLOC_038910 | lncRNA | 46.38295 | 1.966963 | -4.55955 | 3.78E-11 | 6.71E-09 |
| TCONS_00066585 | XLOC_034172 | lncRNA | 41.692909 | 0.350565 | -6.89398 | 4.76E-11 | 8.40E-09 |
| TCONS_00136580 | XLOC_070220 | lncRNA | 0.984881 | 31.018084 | 4.97702 | 7.23E-11 | 1.26E-08 |
| TCONS_00018201 | XLOC_009449 | lncRNA | 0.297258 | 30.275948 | 6.67031 | 1.56E-10 | 2.67E-08 |
| TCONS_00085780 | XLOC_044001 | lncRNA | 39.791584 | 0.311778 | -6.9958 | 1.58E-10 | 2.67E-08 |
| TCONS_00091380 | XLOC_046872 | lncRNA | 39.775681 | 0.363001 | -6.77577 | 1.58E-10 | 2.67E-08 |
| TCONS_00135156 | XLOC_069509 | lncRNA | 1.192818 | 33.071674 | 4.79315 | 2.15E-10 | 3.56E-08 |
| TCONS_00206157 | XLOC_105598 | lncRNA | 1.153097 | 33.413849 | 4.85686 | 2.15E-10 | 3.56E-08 |
| TCONS_00178993 | XLOC_091766 | lncRNA | 38.214787 | 0.337259 | -6.82413 | 2.87E-10 | 4.69E-08 |
| XR_001653388.1 | LOC107809337 | lncRNA | 0.711095 | 29.457691 | 5.37246 | 3.37E-10 | 5.50E-08 |
| TCONS_00000828 | XLOC_000444 | lncRNA | 46.710625 | 2.239621 | -4.38242 | 3.62E-10 | 5.84E-08 |
| TCONS_00049876 | XLOC_025616 | lncRNA | 37.680176 | 0.616329 | -5.93396 | 5.22E-10 | 8.26E-08 |
| TCONS_00018723 | XLOC_009694 | lncRNA | 0.738425 | 28.00181 | 5.24492 | 7.28E-10 | 1.14E-07 |
| TCONS_00035156 | XLOC_018095 | lncRNA | 36.851814 | 0.304679 | -6.9183 | 9.49E-10 | 1.44E-07 |
| TCONS_00213690 | XLOC_109393 | lncRNA | 36.738022 | 0.273369 | -7.07028 | 9.49E-10 | 1.44E-07 |
| TCONS_00180802 | XLOC_092736 | lncRNA | 36.907749 | 0.578183 | -5.99625 | 9.49E-10 | 1.44E-07 |
| TCONS_00109485 | XLOC_056163 | lncRNA | 40.273796 | 1.402942 | -4.84331 | 1.22E-09 | 1.84E-07 |
| TCONS_00136579 | XLOC_070220 | lncRNA | 0.533028 | 27.691446 | 5.69909 | 1.57E-09 | 2.33E-07 |
| TCONS_00181427 | XLOC_093031 | lncRNA | 0.351163 | 27.045853 | 6.26712 | 1.57E-09 | 2.33E-07 |
| TCONS_00042912 | XLOC_022090 | lncRNA | 35.156517 | 0.325959 | -6.75296 | 1.72E-09 | 2.55E-07 |
| TCONS_00133021 | XLOC_068463 | lncRNA | 34.760639 | 0.317538 | -6.77438 | 3.13E-09 | 4.52E-07 |
| TCONS_00158907 | XLOC_081625 | lncRNA | 27.167215 | 0 | #NAME? | 5.14E-09 | 7.25E-07 |
| XR_001654277.1 | LOC107813248 | lncRNA | 128.98349 | 40.377586 | -1.67556 | 6.24E-09 | 8.78E-07 |
| TCONS_00161855 | XLOC_083106 | lncRNA | 0.359247 | 25.396164 | 6.14349 | 7.30E-09 | 1.02E-06 |
| TCONS_00238311 | XLOC_121942 | lncRNA | 0.551021 | 25.157719 | 5.51275 | 7.30E-09 | 1.02E-06 |
| TCONS_00212894 | XLOC_108967 | lncRNA | 47.042446 | 4.017545 | -3.54958 | 8.15E-09 | 1.13E-06 |
| TCONS_00108321 | XLOC_055549 | lncRNA | 0 | 20.641726 | inf | 9.49E-09 | 1.30E-06 |
| TCONS_00096622 | XLOC_049591 | lncRNA | 4.763403 | 35.431499 | 2.89497 | 1.42E-08 | 1.92E-06 |
| TCONS_00042913 | XLOC_022090 | lncRNA | 0.369267 | 24.127298 | 6.02986 | 1.57E-08 | 2.12E-06 |
| TCONS_00128360 | XLOC_066153 | lncRNA | 31.420996 | 0.299199 | -6.71448 | 1.88E-08 | 2.50E-06 |
| TCONS_00110670 | XLOC_056741 | lncRNA | 9.312085 | 44.415192 | 2.25388 | 3.12E-08 | 4.04E-06 |
| XR_001655759.1 | LOC107819642 | lncRNA | 24.505186 | 0 | #NAME? | 3.38E-08 | 4.34E-06 |
| TCONS_00187434 | XLOC_096062 | lncRNA | 30.243389 | 0.261683 | -6.85266 | 3.41E-08 | 4.34E-06 |
| TCONS_00072191 | XLOC_037045 | lncRNA | 29.115629 | 0.265964 | -6.77442 | 6.18E-08 | 7.66E-06 |
| TCONS_00236315 | XLOC_120902 | lncRNA | 29.229498 | 0.277016 | -6.72131 | 6.18E-08 | 7.66E-06 |
| TCONS_00032869 | XLOC_016949 | lncRNA | 29.925365 | 0.297449 | -6.65258 | 6.18E-08 | 7.66E-06 |
| TCONS_00118452 | XLOC_060778 | lncRNA | 0.65669 | 22.085518 | 5.07174 | 7.29E-08 | 8.93E-06 |
| TCONS_00064136 | XLOC_032880 | lncRNA | 0.554241 | 22.669968 | 5.35413 | 7.29E-08 | 8.93E-06 |
| TCONS_00157373 | XLOC_080873 | lncRNA | 0.385176 | 22.360891 | 5.85932 | 7.29E-08 | 8.93E-06 |
| TCONS_00037360 | XLOC_019233 | lncRNA | 1.446612 | 25.208401 | 4.12315 | 7.96E-08 | 9.70E-06 |
| TCONS_00075532 | XLOC_038842 | lncRNA | 28.232069 | 0.312232 | -6.49857 | 1.12E-07 | 1.33E-05 |
| TCONS_00115946 | XLOC_059491 | lncRNA | 28.362051 | 0.267561 | -6.72795 | 1.12E-07 | 1.33E-05 |
| TCONS_00130237 | XLOC_067104 | lncRNA | 2.332373 | 27.008442 | 3.53354 | 1.30E-07 | 1.53E-05 |
| TCONS_00076754 | XLOC_039462 | lncRNA | 0.485846 | 21.34688 | 5.45738 | 1.57E-07 | 1.84E-05 |
| TCONS_00220356 | XLOC_112768 | lncRNA | 1.456081 | 24.959084 | 4.0994 | 1.66E-07 | 1.94E-05 |
| TCONS_00063571 | XLOC_032594 | lncRNA | 27.897558 | 0.306642 | -6.50744 | 2.03E-07 | 2.36E-05 |
| TCONS_00104566 | XLOC_053753 | lncRNA | 2.240537 | 26.273125 | 3.55167 | 2.62E-07 | 3.00E-05 |
| TCONS_00198694 | XLOC_101745 | lncRNA | 0.575399 | 20.845926 | 5.17906 | 3.37E-07 | 3.79E-05 |
| TCONS_00177911 | XLOC_091167 | lncRNA | 0.448136 | 20.697704 | 5.52939 | 3.37E-07 | 3.79E-05 |
| TCONS_00039178 | XLOC_020176 | lncRNA | 26.320183 | 0.384154 | -6.09834 | 3.68E-07 | 4.09E-05 |
| TCONS_00188597 | XLOC_096611 | lncRNA | 26.650614 | 0.468507 | -5.82995 | 3.68E-07 | 4.09E-05 |
| TCONS_00227698 | XLOC_116492 | lncRNA | 30.588371 | 1.02047 | -4.90568 | 3.71E-07 | 4.11E-05 |
| TCONS_00161715 | XLOC_083027 | lncRNA | 25.908085 | 0.419957 | -5.94702 | 6.66E-07 | 7.18E-05 |
| TCONS_00243999 | XLOC_124825 | lncRNA | 1.176148 | 22.634281 | 4.26637 | 7.13E-07 | 7.64E-05 |
| TCONS_00124735 | XLOC_064134 | lncRNA | 0 | 14.222094 | inf | 1.23E-06 | 0.00012781 |
| TCONS_00131504 | XLOC_067736 | lncRNA | 31.987223 | 2.15207 | -3.8937 | 1.43E-06 | 0.00014708 |
| TCONS_00143801 | XLOC_073904 | lncRNA | 1.068701 | 21.002077 | 4.2966 | 1.47E-06 | 0.000151511 |
| TCONS_00182426 | XLOC_093539 | lncRNA | 0.579804 | 18.928278 | 5.02883 | 1.55E-06 | 0.000156182 |
| TCONS_00135473 | XLOC_069669 | lncRNA | 10.61061 | 39.116119 | 1.88226 | 1.64E-06 | 0.000164866 |
| TCONS_00192802 | XLOC_098719 | lncRNA | 23.596273 | 0.303117 | -6.28254 | 2.18E-06 | 0.000215527 |
| TCONS_00219293 | XLOC_112227 | lncRNA | 23.952028 | 0.584643 | -5.35645 | 2.18E-06 | 0.000215527 |
| TCONS_00078901 | XLOC_040479 | lncRNA | 23.631416 | 0.535969 | -5.46241 | 2.18E-06 | 0.000215527 |
| TCONS_00109105 | XLOC_055944 | lncRNA | 0.366662 | 17.311947 | 5.56117 | 3.32E-06 | 0.000320154 |
| TCONS_00096112 | XLOC_049296 | lncRNA | 22.946823 | 0.507387 | -5.49906 | 3.94E-06 | 0.000372031 |
| TCONS_00036613 | XLOC_018858 | lncRNA | 22.955145 | 0.328591 | -6.12638 | 3.94E-06 | 0.000372031 |
| TCONS_00054243 | XLOC_027759 | lncRNA | 2.551331 | 22.661503 | 3.15092 | 4.27E-06 | 0.000399773 |
| XR_001653389.1 | LOC107809337 | lncRNA | 16.389738 | 0 | #NAME? | 5.30E-06 | 0.000489077 |
| TCONS_00004760 | XLOC_002509 | lncRNA | 0.647384 | 16.928663 | 4.7087 | 7.11E-06 | 0.000633566 |
| XR_001644669.1 | LOC107770691 | lncRNA | 21.417782 | 0.42297 | -5.66211 | 7.11E-06 | 0.000633566 |
| XR_001656640.1 | LOC107823449 | lncRNA | 8.085292 | 32.720146 | 2.01681 | 1.24E-05 | 0.001064812 |
| TCONS_00165910 | XLOC_085157 | lncRNA | 1.360586 | 18.485924 | 3.76413 | 1.28E-05 | 0.001087986 |
| TCONS_00147926 | XLOC_075981 | lncRNA | 0 | 11.073429 | inf | 1.43E-05 | 0.001195426 |
| TCONS_00052115 | XLOC_026714 | lncRNA | 0.431937 | 15.371585 | 5.1533 | 1.52E-05 | 0.001258227 |
| TCONS_00099767 | XLOC_051251 | lncRNA | 0.6301 | 15.255277 | 4.59758 | 1.52E-05 | 0.001258227 |
| TCONS_00222593 | XLOC_113940 | lncRNA | 0.421528 | 15.431422 | 5.1941 | 1.52E-05 | 0.001258227 |
| TCONS_00220316 | XLOC_112742 | lncRNA | 3.146605 | 22.249447 | 2.8219 | 1.88E-05 | 0.001535677 |
| TCONS_00093910 | XLOC_048152 | lncRNA | 23.082804 | 1.154204 | -4.32185 | 1.89E-05 | 0.001535677 |
| TCONS_00128615 | XLOC_066291 | lncRNA | 14.027612 | 0 | #NAME? | 1.90E-05 | 0.001535677 |
| TCONS_00204344 | XLOC_104676 | lncRNA | 19.357361 | 0.525212 | -5.20384 | 2.32E-05 | 0.001848142 |
| TCONS_00135293 | XLOC_069560 | lncRNA | 19.048605 | 0.370903 | -5.6825 | 2.32E-05 | 0.001848142 |
| TCONS_00109968 | XLOC_056408 | lncRNA | 19.577673 | 0.279353 | -6.13098 | 2.32E-05 | 0.001848142 |
| TCONS_00066702 | XLOC_034237 | lncRNA | 19.283508 | 0.293911 | -6.03584 | 2.32E-05 | 0.001848142 |
| TCONS_00220521 | XLOC_112844 | lncRNA | 0.493925 | 14.555004 | 4.88108 | 3.24E-05 | 0.002551998 |
| TCONS_00234580 | XLOC_119982 | lncRNA | 25.820833 | 2.034516 | -3.66578 | 3.52E-05 | 0.002720695 |
| TCONS_00061576 | XLOC_031603 | lncRNA | 18.5564 | 0.394325 | -5.55639 | 4.17E-05 | 0.00316211 |
| TCONS_00024079 | XLOC_012400 | lncRNA | 18.277702 | 0.789611 | -4.5328 | 4.17E-05 | 0.00316211 |
| TCONS_00123004 | XLOC_063129 | lncRNA | 1.370538 | 16.510365 | 3.59056 | 5.35E-05 | 0.003998406 |
| TCONS_00175058 | XLOC_089704 | lncRNA | 1.214299 | 16.494165 | 3.76376 | 5.35E-05 | 0.003998406 |
| TCONS_00074636 | XLOC_038362 | lncRNA | 0.479259 | 13.991085 | 4.86756 | 6.92E-05 | 0.005001439 |
| TCONS_00189220 | XLOC_096934 | lncRNA | 0.536628 | 13.585876 | 4.66204 | 6.92E-05 | 0.005001439 |
| TCONS_00026503 | XLOC_013654 | lncRNA | 0.339757 | 13.581511 | 5.321 | 6.92E-05 | 0.005001439 |
| TCONS_00073141 | XLOC_037549 | lncRNA | 0.371638 | 13.149584 | 5.14498 | 6.92E-05 | 0.005001439 |
| TCONS_00154642 | XLOC_079434 | lncRNA | 0.42283 | 13.410581 | 4.98715 | 6.92E-05 | 0.005001439 |
| XR_001648312.1 | LOC107787127 | lncRNA | 55.187836 | 16.168856 | -1.77113 | 7.42E-05 | 0.005288986 |
| TCONS_00109654 | XLOC_056259 | lncRNA | 0 | 9.896797 | inf | 7.47E-05 | 0.005288986 |
| TCONS_00142514 | XLOC_073290 | lncRNA | 17.432802 | 0.406817 | -5.42128 | 7.51E-05 | 0.005288986 |
| TCONS_00144305 | XLOC_074145 | lncRNA | 17.038565 | 0.327906 | -5.69938 | 7.51E-05 | 0.005288986 |
| TCONS_00060735 | XLOC_031178 | lncRNA | 17.764515 | 0.287517 | -5.94921 | 7.51E-05 | 0.005288986 |
| TCONS_00076836 | XLOC_039505 | lncRNA | 17.657764 | 0.319879 | -5.78663 | 7.51E-05 | 0.005288986 |
| TCONS_00080559 | XLOC_041323 | lncRNA | 17.062788 | 0.566807 | -4.91185 | 7.51E-05 | 0.005288986 |
| TCONS_00172922 | XLOC_088660 | lncRNA | 17.184706 | 0.336297 | -5.67525 | 7.51E-05 | 0.005288986 |
| TCONS_00213182 | XLOC_109119 | lncRNA | 20.485987 | 1.017523 | -4.3315 | 9.94E-05 | 0.006958977 |
| TCONS_00195321 | XLOC_099997 | lncRNA | 11.203474 | 0 | #NAME? | 0.000130857 | 0.008950824 |
| XR_001657662.1 | LOC107827991 | lncRNA | 16.991159 | 0.502894 | -5.07839 | 0.000135077 | 0.009113979 |
| TCONS_00125328 | XLOC_064499 | lncRNA | 16.804127 | 0.562992 | -4.89956 | 0.000135077 | 0.009113979 |
| TCONS_00138920 | XLOC_071458 | lncRNA | 16.082447 | 0.301528 | -5.73705 | 0.000135077 | 0.009113979 |
| TCONS_00091284 | XLOC_046818 | lncRNA | 16.839977 | 0.453287 | -5.21532 | 0.000135077 | 0.009113979 |
| TCONS_00159286 | XLOC_081803 | lncRNA | 0.330675 | 12.508684 | 5.24137 | 0.000147199 | 0.009780493 |
| TCONS_00008476 | XLOC_004409 | lncRNA | 0.51824 | 12.173018 | 4.55392 | 0.000147199 | 0.009780493 |
| TCONS_00053862 | XLOC_027574 | lncRNA | 0.366957 | 12.011585 | 5.03267 | 0.000147199 | 0.009780493 |
| TCONS_00083977 | XLOC_043104 | lncRNA | 0.416101 | 12.845419 | 4.94818 | 0.000147199 | 0.009780493 |
| TCONS_00073608 | XLOC_037779 | lncRNA | 0.414769 | 12.834119 | 4.95153 | 0.000147199 | 0.009780493 |
| TCONS_00118306 | XLOC_060709 | lncRNA | 0.192156 | 12.436657 | 6.01618 | 0.000147199 | 0.009780493 |
| TCONS_00206375 | XLOC_105718 | lncRNA | 0.008698 | 12.615947 | 10.5023 | 0.000147199 | 0.009780493 |
| TCONS_00054839 | XLOC_028087 | lncRNA | 0.506094 | 12.801105 | 4.66072 | 0.000147199 | 0.009780493 |
| TCONS_00122596 | XLOC_062924 | lncRNA | 0 | 8.632803 | inf | 0.00017164 | 0.011077162 |
| TCONS_00135760 | XLOC_069830 | lncRNA | 1.075626 | 14.797252 | 3.78208 | 0.000219767 | 0.014036186 |
| XR_001643636.1 | LOC107766229 | lncRNA | 15.814595 | 0.085941 | -7.52369 | 0.000242643 | 0.015152528 |
| TCONS_00215207 | XLOC_110162 | lncRNA | 15.267694 | 0.274073 | -5.79978 | 0.000242643 | 0.015152528 |
| TCONS_00126197 | XLOC_064988 | lncRNA | 15.040365 | 0.427076 | -5.1382 | 0.000242643 | 0.015152528 |
| TCONS_00213133 | XLOC_109096 | lncRNA | 15.516888 | 0.302366 | -5.6814 | 0.000242643 | 0.015152528 |
| TCONS_00170376 | XLOC_087402 | lncRNA | 15.14902 | 0.067063 | -7.81949 | 0.000242643 | 0.015152528 |
| TCONS_00109978 | XLOC_056409 | lncRNA | 15.481167 | 0.465864 | -5.05446 | 0.000242643 | 0.015152528 |
| TCONS_00161852 | XLOC_083107 | lncRNA | 15.262953 | 0.12597 | -6.92081 | 0.000242643 | 0.015152528 |
| TCONS_00096892 | XLOC_049727 | lncRNA | 15.260865 | 0.183062 | -6.38136 | 0.000242643 | 0.015152528 |
| TCONS_00079615 | XLOC_040833 | lncRNA | 15.07605 | 0.427298 | -5.14087 | 0.000242643 | 0.015152528 |
| TCONS_00007464 | XLOC_003897 | lncRNA | 15.499187 | 0.288576 | -5.7471 | 0.000242643 | 0.015152528 |
| TCONS_00065534 | XLOC_033612 | lncRNA | 18.464489 | 1.85736 | -3.31343 | 0.000296559 | 0.018065173 |
| TCONS_00144870 | XLOC_074409 | lncRNA | 0.955786 | 11.203272 | 3.55109 | 0.000312662 | 0.018745522 |
| TCONS_00073938 | XLOC_037971 | lncRNA | 0.426003 | 11.90669 | 4.80477 | 0.000312662 | 0.018745522 |
| TCONS_00202643 | XLOC_103794 | lncRNA | 0.460696 | 11.16059 | 4.59845 | 0.000312662 | 0.018745522 |
| TCONS_00020708 | XLOC_010669 | lncRNA | 0.876998 | 11.852836 | 3.75651 | 0.000312662 | 0.018745522 |
| TCONS_00000113 | XLOC_000054 | lncRNA | 0.636017 | 11.047539 | 4.11852 | 0.000312662 | 0.018745522 |
| TCONS_00186012 | XLOC_095355 | lncRNA | 0.500511 | 11.888574 | 4.57003 | 0.000312662 | 0.018745522 |
| TCONS_00057003 | XLOC_029202 | lncRNA | 0.523376 | 11.896461 | 4.50654 | 0.000312662 | 0.018745522 |
| TCONS_00152950 | XLOC_078549 | lncRNA | 0.358965 | 11.591278 | 5.01305 | 0.000312662 | 0.018745522 |
| TCONS_00043571 | XLOC_022385 | lncRNA | 37.337116 | 9.392025 | -1.9911 | 0.000330926 | 0.019803698 |
| XR_001647513.1 | LOC107783772 | lncRNA | 60.829407 | 21.856422 | -1.47671 | 0.000331555 | 0.01982297 |
| XR_001658623.1 | LOC107832699 | lncRNA | 0 | 7.863518 | inf | 0.000396238 | 0.023091572 |
| TCONS_00024696 | XLOC_012731 | lncRNA | 14.582489 | 0.246411 | -5.88703 | 0.000435319 | 0.025097334 |
| TCONS_00101988 | XLOC_052432 | lncRNA | 14.205366 | 0.353333 | -5.32926 | 0.000435319 | 0.025097334 |
| TCONS_00155864 | XLOC_080064 | lncRNA | 14.418707 | 0.35573 | -5.34102 | 0.000435319 | 0.025097334 |
| TCONS_00225668 | XLOC_115512 | lncRNA | 14.068215 | 0.423813 | -5.05287 | 0.000435319 | 0.025097334 |
| TCONS_00114274 | XLOC_058641 | lncRNA | 9.511714 | 0 | #NAME? | 0.000480164 | 0.02707829 |
| XR_001644220.1 | LOC107768727 | lncRNA | 17.94216 | 1.100759 | -4.02678 | 0.000510136 | 0.028445614 |
| TCONS_00023143 | XLOC_011922 | lncRNA | 17.671022 | 1.62962 | -3.43878 | 0.000510136 | 0.028445614 |
| TCONS_00039285 | XLOC_020249 | lncRNA | 0.352483 | 10.517319 | 4.89907 | 0.000662682 | 0.036386096 |
| TCONS_00213871 | XLOC_109490 | lncRNA | 0.642404 | 10.834083 | 4.07595 | 0.000662682 | 0.036386096 |
| TCONS_00095804 | XLOC_049149 | lncRNA | 0.497691 | 10.225545 | 4.36078 | 0.000662682 | 0.036386096 |
| TCONS_00169330 | XLOC_086891 | lncRNA | 0.356967 | 10.636135 | 4.89704 | 0.000662682 | 0.036386096 |
| XR_001646008.1 | LOC107776602 | lncRNA | 43.681862 | 13.908998 | -1.65102 | 0.000698108 | 0.038136683 |
| XR_001649453.1 | LOC107792131 | lncRNA | 41.702946 | 12.490816 | -1.73928 | 0.000717759 | 0.039110882 |
| TCONS_00052965 | XLOC_027123 | lncRNA | 13.431649 | 0.579447 | -4.53482 | 0.000779902 | 0.041735151 |
| TCONS_00177085 | XLOC_090752 | lncRNA | 13.737588 | 0.287628 | -5.57778 | 0.000779902 | 0.041735151 |
| TCONS_00110323 | XLOC_056578 | lncRNA | 13.593595 | 0.288244 | -5.55949 | 0.000779902 | 0.041735151 |
| TCONS_00014874 | XLOC_007704 | lncRNA | 13.833717 | 0.94892 | -3.86576 | 0.000779902 | 0.041735151 |
| TCONS_00146715 | XLOC_075386 | lncRNA | 13.142027 | 0.294156 | -5.48146 | 0.000779902 | 0.041735151 |
| TCONS_00072006 | XLOC_036938 | lncRNA | 13.740651 | 0.266415 | -5.68863 | 0.000779902 | 0.041735151 |
| TCONS_00165917 | XLOC_085161 | lncRNA | 13.653818 | 0.289779 | -5.55821 | 0.000779902 | 0.041735151 |
| TCONS_00182966 | XLOC_093810 | lncRNA | 13.049777 | 0.976253 | -3.74063 | 0.000779902 | 0.041735151 |
| TCONS_00001525 | XLOC_000781 | lncRNA | 0 | 6.134433 | inf | 0.000920448 | 0.047393603 |
| XR_001643236.1 | LOC107764415 | lncRNA | 8.275765 | 0 | #NAME? | 0.000924964 | 0.047393603 |
