# Supplementary material for: Genome-wide identification and functional prediction of tobacco lncRNAs responsive to root-knot nematode stress
Source: PLoS One. 2018 Nov 14;13(11):e0204506. doi: 10.1371/journal.pone.0204506 (PMC6235259; doi:10.1371/journal.pone.0204506)
Supplement: S4 Table — (DOCX) [file pone.0204506.s006.docx]

**S4 Table. Gene ontology classifcation of target transcripts for all diﬀerentially expressed lncRNAs in G28.**

| category | over_represented_pvalue | under_represented_pvalue | numDEInCat | numInCat | term | ontology |
| --- | --- | --- | --- | --- | --- | --- |
| GO:0004652 | 0.000335468 | 0.999997677 | 2 | 10 | polynucleotide adenylyltransferase activity | Molecular Function |
| GO:0004564 | 0.003933244 | 0.999996268 | 1 | 2 | beta-fructofuranosidase activity | Molecular Function |
| GO:0004536 | 0.005797199 | 0.999989184 | 1 | 3 | deoxyribonuclease activity | Molecular Function |
| GO:0009678 | 0.009686168 | 0.999963718 | 1 | 5 | hydrogen-translocating pyrophosphatase activity | Molecular Function |
| GO:0035673 | 0.00981595 | 0.999962737 | 1 | 5 | oligopeptide transmembrane transporter activity | Molecular Function |
| GO:0016888 | 0.011539377 | 0.999946327 | 1 | 6 | endodeoxyribonuclease activity, producing 5'-phosphomonoesters | Molecular Function |
| GO:0008374 | 0.017519777 | 0.999867751 | 1 | 9 | O-acyltransferase activity | Molecular Function |
| GO:0004575 | 0.01755084 | 0.99986728 | 1 | 9 | sucrose alpha-glucosidase activity | Molecular Function |
| GO:0004869 | 0.020524698 | 0.99981418 | 1 | 11 | cysteine-type endopeptidase inhibitor activity | Molecular Function |
| GO:0008143 | 0.022978023 | 0.999766883 | 1 | 11 | poly(A) binding | Molecular Function |
| GO:0004427 | 0.024776008 | 0.999724648 | 1 | 13 | inorganic diphosphatase activity | Molecular Function |
| GO:0004620 | 0.029485989 | 0.999605011 | 1 | 15 | phospholipase activity | Molecular Function |
| GO:0009982 | 0.04225493 | 0.999166649 | 1 | 22 | pseudouridine synthase activity | Molecular Function |
| GO:0004518 | 0.042705709 | 0.999148631 | 1 | 22 | nuclease activity | Molecular Function |
| GO:0046872 | 0.05013352 | 0.982812323 | 7 | 1701 | metal ion binding | Molecular Function |
| GO:0016853 | 0.051366512 | 0.998757368 | 1 | 25 | isomerase activity | Molecular Function |
| GO:0000149 | 0.053218381 | 0.998659382 | 1 | 28 | SNARE binding | Molecular Function |
| GO:0003723 | 0.058415094 | 0.98278954 | 5 | 1028 | RNA binding | Molecular Function |
| GO:0005484 | 0.062110424 | 0.998157836 | 1 | 33 | SNAP receptor activity | Molecular Function |
| GO:0043531 | 0.096829215 | 0.995423028 | 1 | 47 | ADP binding | Molecular Function |
| GO:0003887 | 0.119356538 | 0.992962185 | 1 | 56 | DNA-directed DNA polymerase activity | Molecular Function |
| GO:0016740 | 0.123198679 | 0.992469305 | 1 | 67 | transferase activity | Molecular Function |
| GO:0004601 | 0.142415573 | 0.989839717 | 1 | 79 | peroxidase activity | Molecular Function |
| GO:0016757 | 0.176054566 | 0.984229859 | 1 | 98 | transferase activity, transferring glycosyl groups | Molecular Function |
| GO:0004519 | 0.224858722 | 0.941273893 | 2 | 425 | endonuclease activity | Molecular Function |
| GO:0003729 | 0.28238673 | 0.957439179 | 1 | 164 | mRNA binding | Molecular Function |
| GO:0020037 | 0.338704293 | 0.937144875 | 1 | 211 | heme binding | Molecular Function |
| GO:0004842 | 0.344436649 | 0.934830165 | 1 | 210 | ubiquitin-protein transferase activity | Molecular Function |
| GO:0003735 | 0.351839192 | 0.931748399 | 1 | 225 | structural constituent of ribosome | Molecular Function |
| GO:0043565 | 0.503601242 | 0.848960949 | 1 | 346 | sequence-specific DNA binding | Molecular Function |
| GO:0003700 | 0.504665488 | 0.767840562 | 2 | 853 | DNA binding transcription factor activity | Molecular Function |
| GO:0008270 | 0.619020336 | 0.755655433 | 1 | 469 | zinc ion binding | Molecular Function |
| GO:0003677 | 0.620231214 | 0.661033973 | 2 | 1004 | DNA binding | Molecular Function |
| GO:0005524 | 0.9016806 | 0.263464735 | 2 | 1731 | ATP binding | Molecular Function |
| GO:0031201 | 0.060259742 | 0.99826883 | 1 | 32 | SNARE complex | Cellular Component |
| GO:0009707 | 0.074751105 | 0.997308332 | 1 | 38 | chloroplast outer membrane | Cellular Component |
| GO:0009705 | 0.13762524 | 0.990535466 | 1 | 75 | plant-type vacuole membrane | Cellular Component |
| GO:0031965 | 0.152545615 | 0.988308475 | 1 | 73 | nuclear membrane | Cellular Component |
| GO:0043231 | 0.204134448 | 0.949852009 | 2 | 398 | intracellular membrane-bounded organelle | Cellular Component |
| GO:0009505 | 0.207855997 | 0.977697581 | 1 | 119 | plant-type cell wall | Cellular Component |
| GO:0005840 | 0.225870522 | 0.973447366 | 1 | 133 | ribosome | Cellular Component |
| GO:0016021 | 0.22979083 | 0.883537809 | 7 | 2505 | integral component of membrane | Cellular Component |
| GO:0005887 | 0.250929399 | 0.966865747 | 1 | 145 | integral component of plasma membrane | Cellular Component |
| GO:0005576 | 0.25511448 | 0.927706212 | 2 | 504 | extracellular region | Cellular Component |
| GO:0048046 | 0.325044461 | 0.942481066 | 1 | 201 | apoplast | Cellular Component |
| GO:0005737 | 0.363476846 | 0.804200258 | 5 | 1953 | cytoplasm | Cellular Component |
| GO:0016020 | 0.372568515 | 0.863455564 | 2 | 629 | membrane | Cellular Component |
| GO:0005618 | 0.446304661 | 0.884934459 | 1 | 300 | cell wall | Cellular Component |
| GO:0005654 | 0.458755801 | 0.877662924 | 1 | 286 | nucleoplasm | Cellular Component |
| GO:0000139 | 0.469930683 | 0.870850179 | 1 | 315 | Golgi membrane | Cellular Component |
| GO:0005634 | 0.482405375 | 0.686282731 | 7 | 3178 | nucleus | Cellular Component |
| GO:0005783 | 0.594130797 | 0.778408378 | 1 | 436 | endoplasmic reticulum | Cellular Component |
| GO:0009506 | 0.594897868 | 0.777755333 | 1 | 409 | plasmodesma | Cellular Component |
| GO:0005886 | 0.615702691 | 0.621553885 | 3 | 1520 | plasma membrane | Cellular Component |
| GO:0005794 | 0.652704094 | 0.72226042 | 1 | 507 | Golgi apparatus | Cellular Component |
| GO:0005829 | 0.7550606 | 0.501761215 | 2 | 1284 | cytosol | Cellular Component |
| GO:0005739 | 0.824157674 | 0.491868916 | 1 | 826 | mitochondrion | Cellular Component |
| GO:0035722 | 0.001873261 | 1 | 1 | 1 | interleukin-12-mediated signaling pathway | Biological Process |
| GO:0030509 | 0.003810235 | 0.999996498 | 1 | 2 | BMP signaling pathway | Biological Process |
| GO:0034393 | 0.003810235 | 0.999996498 | 1 | 2 | positive regulation of smooth muscle cell apoptotic process | Biological Process |
| GO:0050729 | 0.003810235 | 0.999996498 | 1 | 2 | positive regulation of inflammatory response | Biological Process |
| GO:0060940 | 0.003810235 | 0.999996498 | 1 | 2 | epithelial to mesenchymal transition involved in cardiac fibroblast development | Biological Process |
| GO:1901224 | 0.003810235 | 0.999996498 | 1 | 2 | positive regulation of NIK/NF-kappaB signaling | Biological Process |
| GO:1904761 | 0.003810235 | 0.999996498 | 1 | 2 | negative regulation of myofibroblast differentiation | Biological Process |
| GO:1905064 | 0.003810235 | 0.999996498 | 1 | 2 | negative regulation of vascular smooth muscle cell differentiation | Biological Process |
| GO:2000353 | 0.003810235 | 0.999996498 | 1 | 2 | positive regulation of endothelial cell apoptotic process | Biological Process |
| GO:1900016 | 0.005725414 | 0.999989451 | 1 | 3 | negative regulation of cytokine production involved in inflammatory response | Biological Process |
| GO:0043508 | 0.005774999 | 0.999989267 | 1 | 3 | negative regulation of JUN kinase activity | Biological Process |
| GO:0045786 | 0.005794133 | 0.999989196 | 1 | 3 | negative regulation of cell cycle | Biological Process |
| GO:1990481 | 0.007841296 | 0.999977723 | 1 | 4 | mRNA pseudouridine synthesis | Biological Process |
| GO:0043630 | 0.00835382 | 0.999983128 | 1 | 2 | ncRNA polyadenylation involved in polyadenylation-dependent ncRNA catabolic process | Biological Process |
| GO:0006397 | 0.009228375 | 0.999120119 | 3 | 212 | mRNA processing | Biological Process |
| GO:0046786 | 0.009603818 | 0.999964333 | 1 | 5 | viral replication complex formation and maintenance | Biological Process |
| GO:0009682 | 0.01152003 | 0.999946507 | 1 | 6 | induced systemic resistance | Biological Process |
| GO:0006308 | 0.011586182 | 0.999945889 | 1 | 6 | DNA catabolic process | Biological Process |
| GO:0071222 | 0.011662256 | 0.999945175 | 1 | 6 | cellular response to lipopolysaccharide | Biological Process |
| GO:0007569 | 0.01354827 | 0.999923844 | 1 | 7 | cell aging | Biological Process |
| GO:0009395 | 0.017484475 | 0.999868285 | 1 | 9 | phospholipid catabolic process | Biological Process |
| GO:0042493 | 0.017497864 | 0.99987277 | 1 | 7 | response to drug | Biological Process |
| GO:0031119 | 0.017538111 | 0.999867473 | 1 | 9 | tRNA pseudouridine synthesis | Biological Process |
| GO:0009567 | 0.01918583 | 0.999839328 | 1 | 10 | double fertilization forming a zygote and endosperm | Biological Process |
| GO:0050832 | 0.028221538 | 0.997770576 | 2 | 128 | defense response to fungus | Biological Process |
| GO:0010193 | 0.032685116 | 0.999510026 | 1 | 17 | response to ozone | Biological Process |
| GO:0015992 | 0.034111695 | 0.999464204 | 1 | 18 | proton transport | Biological Process |
| GO:0007062 | 0.038281193 | 0.99933538 | 1 | 14 | sister chromatid cohesion | Biological Process |
| GO:0009414 | 0.039392099 | 0.996266761 | 2 | 158 | response to water deprivation | Biological Process |
| GO:0009409 | 0.040309543 | 0.996130632 | 2 | 163 | response to cold | Biological Process |
| GO:0006906 | 0.045544871 | 0.999026854 | 1 | 24 | vesicle fusion | Biological Process |
| GO:0010628 | 0.049303388 | 0.99885426 | 1 | 26 | positive regulation of gene expression | Biological Process |
| GO:0051865 | 0.051384594 | 0.998752714 | 1 | 27 | protein autoubiquitination | Biological Process |
| GO:0043066 | 0.051509763 | 0.998748394 | 1 | 26 | negative regulation of apoptotic process | Biological Process |
| GO:0006004 | 0.060908548 | 0.998230924 | 1 | 32 | fucose metabolic process | Biological Process |
| GO:0006887 | 0.072731427 | 0.997459489 | 1 | 35 | exocytosis | Biological Process |
| GO:0009737 | 0.075726625 | 0.98967256 | 2 | 220 | response to abscisic acid | Biological Process |
| GO:0006915 | 0.079711385 | 0.996933565 | 1 | 38 | apoptotic process | Biological Process |
| GO:0009651 | 0.082876 | 0.988098915 | 2 | 241 | response to salt stress | Biological Process |
| GO:0016567 | 0.083536513 | 0.987949829 | 2 | 241 | protein ubiquitination | Biological Process |
| GO:0080167 | 0.096704161 | 0.995425842 | 1 | 52 | response to karrikin | Biological Process |
| GO:0015031 | 0.104777284 | 0.982780111 | 2 | 264 | protein transport | Biological Process |
| GO:0042744 | 0.121909832 | 0.992629685 | 1 | 67 | hydrogen peroxide catabolic process | Biological Process |
| GO:0009626 | 0.127209907 | 0.99196402 | 1 | 64 | plant-type hypersensitive response | Biological Process |
| GO:0006970 | 0.151949758 | 0.98838731 | 1 | 81 | response to osmotic stress | Biological Process |
| GO:0009735 | 0.153444683 | 0.988144186 | 1 | 85 | response to cytokinin | Biological Process |
| GO:0009451 | 0.160127169 | 0.96614252 | 2 | 338 | RNA modification | Biological Process |
| GO:0006486 | 0.161452191 | 0.986826604 | 1 | 89 | protein glycosylation | Biological Process |
| GO:0045892 | 0.186427361 | 0.982241773 | 1 | 99 | negative regulation of transcription, DNA-templated | Biological Process |
| GO:0009611 | 0.190072787 | 0.981510004 | 1 | 101 | response to wounding | Biological Process |
| GO:0006364 | 0.210357811 | 0.977147043 | 1 | 111 | rRNA processing | Biological Process |
| GO:0006979 | 0.245938602 | 0.968238779 | 1 | 144 | response to oxidative stress | Biological Process |
| GO:0005975 | 0.287046262 | 0.955924245 | 1 | 171 | carbohydrate metabolic process | Biological Process |
| GO:0006412 | 0.332071175 | 0.939766715 | 1 | 209 | translation | Biological Process |
