# Supplementary material for: Genome-wide identification and functional prediction of tobacco lncRNAs responsive to root-knot nematode stress
Source: PLoS One. 2018 Nov 14;13(11):e0204506. doi: 10.1371/journal.pone.0204506 (PMC6235259; doi:10.1371/journal.pone.0204506)
Supplement: S5 Table — (DOCX) [file pone.0204506.s007.docx]

**S5 Table. Gene ontology classifcation of target transcripts for all diﬀerentially expressed lncRNAs in Long bohuang.**

| Category | over_represented  p value | under_represented  p value | numDEIn  Cat | numIn  Cat | Term | Ontology |
| --- | --- | --- | --- | --- | --- | --- |
| GO:0043027 | 0.001831571 | 1 | 1 | 1 | cysteine-type endopeptidase inhibitor activity involved in apoptotic process | Molecular Function |
| GO:0089720 | 0.001831571 | 1 | 1 | 1 | caspase binding | Molecular Function |
| GO:0047453 | 0.001922509 | 1 | 1 | 1 | ATP-dependent NAD(P)H-hydrate dehydratase activity | Molecular Function |
| GO:0052855 | 0.001922509 | 1 | 1 | 1 | ADP-dependent NAD(P)H-hydrate dehydratase activity | Molecular Function |
| GO:0046554 | 0.001930171 | 1 | 1 | 1 | malate dehydrogenase (NADP+) activity | Molecular Function |
| GO:0051745 | 0.001949156 | 1 | 1 | 1 | 4-hydroxy-3-methylbut-2-en-1-yl diphosphate reductase activity | Molecular Function |
| GO:0015662 | 0.002023071 | 1 | 1 | 1 | ATPase activity, coupled to transmembrane movement of ions, phosphorylative mechanism | Molecular Function |
| GO:0042623 | 0.002023071 | 1 | 1 | 1 | ATPase activity, coupled | Molecular Function |
| GO:0019239 | 0.003377525 | 0.999997262 | 1 | 2 | deaminase activity | Molecular Function |
| GO:0052856 | 0.003891913 | 0.999996364 | 1 | 2 | NADHX epimerase activity | Molecular Function |
| GO:0052857 | 0.003891913 | 0.999996364 | 1 | 2 | NADPHX epimerase activity | Molecular Function |
| GO:0046429 | 0.004637757 | 0.999993115 | 1 | 3 | 4-hydroxy-3-methylbut-2-en-1-yl diphosphate synthase activity | Molecular Function |
| GO:0019829 | 0.00745603 | 0.999979958 | 1 | 4 | cation-transporting ATPase activity | Molecular Function |
| GO:0004737 | 0.00747044 | 0.99997988 | 1 | 4 | pyruvate decarboxylase activity | Molecular Function |
| GO:0016810 | 0.00760343 | 0.999979156 | 1 | 4 | hydrolase activity, acting on carbon-nitrogen (but not peptide) bonds | Molecular Function |
| GO:0045548 | 0.008066785 | 0.999976534 | 1 | 4 | phenylalanine ammonia-lyase activity | Molecular Function |
| GO:0051538 | 0.013529217 | 0.999922854 | 1 | 8 | 3 iron, 4 sulfur cluster binding | Molecular Function |
| GO:0030976 | 0.016805588 | 0.999878929 | 1 | 9 | thiamine pyrophosphate binding | Molecular Function |
| GO:0008553 | 0.019439598 | 0.999835821 | 1 | 10 | hydrogen-exporting ATPase activity, phosphorylative mechanism | Molecular Function |
| GO:0005528 | 0.020847802 | 0.999806258 | 1 | 13 | FK506 binding | Molecular Function |
| GO:0004650 | 0.021545904 | 0.999794439 | 1 | 12 | polygalacturonase activity | Molecular Function |
| GO:0015079 | 0.031858661 | 0.999536848 | 1 | 17 | potassium ion transmembrane transporter activity | Molecular Function |
| GO:0004298 | 0.034705667 | 0.999442058 | 1 | 22 | threonine-type endopeptidase activity | Molecular Function |
| GO:0035091 | 0.039698114 | 0.999270269 | 1 | 21 | phosphatidylinositol binding | Molecular Function |
| GO:0000049 | 0.08158014 | 0.996786265 | 1 | 46 | tRNA binding | Molecular Function |
| GO:0003755 | 0.08385661 | 0.996593041 | 1 | 52 | peptidyl-prolyl cis-trans isomerase activity | Molecular Function |
| GO:0016740 | 0.115367096 | 0.993446442 | 1 | 67 | transferase activity | Molecular Function |
| GO:0043565 | 0.127300495 | 0.976682897 | 2 | 346 | sequence-specific DNA binding | Molecular Function |
| GO:0031625 | 0.13405266 | 0.991068161 | 1 | 79 | ubiquitin protein ligase binding | Molecular Function |
| GO:0016874 | 0.139975928 | 0.9902375 | 1 | 80 | ligase activity | Molecular Function |
| GO:0051082 | 0.145987771 | 0.989347098 | 1 | 86 | unfolded protein binding | Molecular Function |
| GO:0004519 | 0.198788276 | 0.952256477 | 2 | 425 | endonuclease activity | Molecular Function |
| GO:0008270 | 0.209314342 | 0.948061466 | 2 | 469 | zinc ion binding | Molecular Function |
| GO:0000287 | 0.214949355 | 0.97616572 | 1 | 134 | magnesium ion binding | Molecular Function |
| GO:0016491 | 0.216464257 | 0.975808686 | 1 | 138 | oxidoreductase activity | Molecular Function |
| GO:0005516 | 0.248420626 | 0.967697111 | 1 | 148 | calmodulin binding | Molecular Function |
| GO:0005506 | 0.255374624 | 0.96574445 | 1 | 162 | iron ion binding | Molecular Function |
| GO:0061630 | 0.274096999 | 0.960192516 | 1 | 185 | ubiquitin protein ligase activity | Molecular Function |
| GO:0003723 | 0.303763182 | 0.877528198 | 3 | 1028 | RNA binding | Molecular Function |
| GO:0004842 | 0.32192553 | 0.943886234 | 1 | 210 | ubiquitin-protein transferase activity | Molecular Function |
| GO:0003700 | 0.451320317 | 0.810536245 | 2 | 853 | DNA binding transcription factor activity | Molecular Function |
| GO:0046872 | 0.607226769 | 0.63219114 | 3 | 1701 | metal ion binding | Molecular Function |
| GO:0005524 | 0.657896576 | 0.578096285 | 3 | 1731 | ATP binding | Molecular Function |
| GO:0045171 | 0.009076672 | 0.999968301 | 1 | 5 | intercellular bridge | Cellular Component |
| GO:0030123 | 0.009182407 | 0.999967557 | 1 | 5 | AP-3 adaptor complex | Cellular Component |
| GO:0019774 | 0.013626688 | 0.999920498 | 1 | 9 | proteasome core complex, beta-subunit complex | Cellular Component |
| GO:0015630 | 0.017266935 | 0.999870578 | 1 | 10 | microtubule cytoskeleton | Cellular Component |
| GO:0009986 | 0.026490624 | 0.999684664 | 1 | 14 | cell surface | Cellular Component |
| GO:0030687 | 0.029954969 | 0.999590838 | 1 | 17 | preribosome, large subunit precursor | Cellular Component |
| GO:0030659 | 0.049183794 | 0.9988636 | 1 | 27 | cytoplasmic vesicle membrane | Cellular Component |
| GO:0005778 | 0.053740689 | 0.998635829 | 1 | 30 | peroxisomal membrane | Cellular Component |
| GO:0009507 | 0.073656837 | 0.977093838 | 5 | 1272 | chloroplast | Cellular Component |
| GO:0048471 | 0.11666033 | 0.993296848 | 1 | 66 | perinuclear region of cytoplasm | Cellular Component |
| GO:0009705 | 0.129571834 | 0.991675159 | 1 | 75 | plant-type vacuole membrane | Cellular Component |
| GO:0005886 | 0.303676201 | 0.860730257 | 4 | 1520 | plasma membrane | Cellular Component |
| GO:0005773 | 0.408059033 | 0.906011071 | 1 | 294 | vacuole | Cellular Component |
| GO:0005618 | 0.411636989 | 0.904181984 | 1 | 300 | cell wall | Cellular Component |
| GO:0005829 | 0.418326043 | 0.799267099 | 3 | 1284 | cytosol | Cellular Component |
| GO:0009570 | 0.479512752 | 0.86533479 | 1 | 354 | chloroplast stroma | Cellular Component |
| GO:0005789 | 0.520468277 | 0.837731497 | 1 | 402 | endoplasmic reticulum membrane | Cellular Component |
| GO:0009506 | 0.527750728 | 0.832470971 | 1 | 409 | plasmodesma | Cellular Component |
| GO:0005634 | 0.529537118 | 0.654515549 | 6 | 3178 | nucleus | Cellular Component |
| GO:0043231 | 0.545613217 | 0.81911634 | 1 | 398 | intracellular membrane-bounded organelle | Cellular Component |
| GO:0005576 | 0.583660737 | 0.788216303 | 1 | 504 | extracellular region | Cellular Component |
| GO:0005794 | 0.615377675 | 0.759885292 | 1 | 507 | Golgi apparatus | Cellular Component |
| GO:0016020 | 0.687222266 | 0.68559189 | 1 | 629 | membrane | Cellular Component |
| GO:0016021 | 0.700382604 | 0.503534371 | 4 | 2505 | integral component of membrane | Cellular Component |
| GO:0005737 | 0.701792097 | 0.527437879 | 3 | 1953 | cytoplasm | Cellular Component |
| GO:0005739 | 0.788107664 | 0.551858019 | 1 | 826 | mitochondrion | Cellular Component |
| GO:0006964 | 0.001831571 | 1 | 1 | 1 | positive regulation of biosynthetic process of antibacterial peptides active against Gram-negative bacteria | Biological Process |
| GO:0061057 | 0.001831571 | 1 | 1 | 1 | peptidoglycan recognition protein signaling pathway | Biological Process |
| GO:1990001 | 0.001831571 | 1 | 1 | 1 | inhibition of cysteine-type endopeptidase activity involved in apoptotic process | Biological Process |
| GO:1902459 | 0.003388204 | 0.999997245 | 1 | 2 | positive regulation of stem cell population maintenance | Biological Process |
| GO:0043281 | 0.003449895 | 0.999997144 | 1 | 2 | regulation of cysteine-type endopeptidase activity involved in apoptotic process | Biological Process |
| GO:0010595 | 0.003705987 | 0.999996703 | 1 | 2 | positive regulation of endothelial cell migration | Biological Process |
| GO:0014909 | 0.003705987 | 0.999996703 | 1 | 2 | smooth muscle cell migration | Biological Process |
| GO:0033160 | 0.003742646 | 0.999996638 | 1 | 2 | positive regulation of protein import into nucleus, translocation | Biological Process |
| GO:0006723 | 0.003841054 | 0.999996458 | 1 | 2 | cuticle hydrocarbon biosynthetic process | Biological Process |
| GO:0006734 | 0.003891913 | 0.999996364 | 1 | 2 | NADH metabolic process | Biological Process |
| GO:0034227 | 0.005164779 | 0.999991459 | 1 | 3 | tRNA thio-modification | Biological Process |
| GO:0043447 | 0.005274191 | 0.999991093 | 1 | 3 | alkane biosynthetic process | Biological Process |
| GO:0043154 | 0.005358056 | 0.999990807 | 1 | 3 | negative regulation of cysteine-type endopeptidase activity involved in apoptotic process | Biological Process |
| GO:0006739 | 0.005618267 | 0.999989891 | 1 | 3 | NADP metabolic process | Biological Process |
| GO:0007423 | 0.005866697 | 0.999988976 | 1 | 3 | sensory organ development | Biological Process |
| GO:0050992 | 0.006711534 | 0.999983766 | 1 | 4 | dimethylallyl diphosphate biosynthetic process | Biological Process |
| GO:0010023 | 0.00688933 | 0.999982893 | 1 | 4 | proanthocyanidin biosynthetic process | Biological Process |
| GO:0001525 | 0.007332387 | 0.999980618 | 1 | 4 | angiogenesis | Biological Process |
| GO:0009800 | 0.00953556 | 0.999965008 | 1 | 5 | cinnamic acid biosynthetic process | Biological Process |
| GO:0070370 | 0.009974947 | 0.999961702 | 1 | 5 | cellular heat acclimation | Biological Process |
| GO:0008610 | 0.010466158 | 0.999956077 | 1 | 6 | lipid biosynthetic process | Biological Process |
| GO:0016558 | 0.01312274 | 0.999928907 | 1 | 7 | protein import into peroxisome matrix | Biological Process |
| GO:0070936 | 0.013236399 | 0.999927667 | 1 | 7 | protein K48-linked ubiquitination | Biological Process |
| GO:0050829 | 0.013311371 | 0.999926843 | 1 | 7 | defense response to Gram-negative bacterium | Biological Process |
| GO:0006754 | 0.013830103 | 0.999921013 | 1 | 7 | ATP biosynthetic process | Biological Process |
| GO:0009870 | 0.015364585 | 0.999898858 | 1 | 9 | defense response signaling pathway, resistance gene-dependent | Biological Process |
| GO:0019288 | 0.015913262 | 0.999891482 | 1 | 9 | isopentenyl diphosphate biosynthetic process, methylerythritol 4-phosphate pathway | Biological Process |
| GO:0009567 | 0.017084956 | 0.9998733 | 1 | 10 | double fertilization forming a zygote and endosperm | Biological Process |
| GO:0006108 | 0.01789056 | 0.999861027 | 1 | 10 | malate metabolic process | Biological Process |
| GO:0010588 | 0.019381668 | 0.999836802 | 1 | 10 | cotyledon vascular tissue pattern formation | Biological Process |
| GO:0006559 | 0.019613152 | 0.999832865 | 1 | 10 | L-phenylalanine catabolic process | Biological Process |
| GO:0009966 | 0.020543951 | 0.999814718 | 1 | 11 | regulation of signal transduction | Biological Process |
| GO:0010078 | 0.021932427 | 0.999786966 | 1 | 12 | maintenance of root meristem identity | Biological Process |
| GO:0010214 | 0.022471339 | 0.999776322 | 1 | 12 | seed coat development | Biological Process |
| GO:0002098 | 0.023070691 | 0.99976254 | 1 | 13 | tRNA wobble uridine modification | Biological Process |
| GO:0042335 | 0.023076994 | 0.99976241 | 1 | 13 | cuticle development | Biological Process |
| GO:1900865 | 0.023765389 | 0.999749695 | 1 | 12 | chloroplast RNA modification | Biological Process |
| GO:0010025 | 0.025852648 | 0.999699742 | 1 | 14 | wax biosynthetic process | Biological Process |
| GO:0007035 | 0.02639492 | 0.999685358 | 1 | 15 | vacuolar acidification | Biological Process |
| GO:0042273 | 0.029450413 | 0.999603221 | 1 | 18 | ribosomal large subunit biogenesis | Biological Process |
| GO:0070534 | 0.029612154 | 0.999600201 | 1 | 17 | protein K63-linked ubiquitination | Biological Process |
| GO:0009835 | 0.032697049 | 0.999510321 | 1 | 18 | fruit ripening | Biological Process |
| GO:0048235 | 0.033740314 | 0.999476773 | 1 | 19 | pollen sperm cell differentiation | Biological Process |
| GO:0007033 | 0.035136961 | 0.999432263 | 1 | 19 | vacuole organization | Biological Process |
| GO:0010305 | 0.036110952 | 0.999400133 | 1 | 19 | leaf vascular tissue pattern formation | Biological Process |
| GO:0005975 | 0.03951415 | 0.996272837 | 2 | 171 | carbohydrate metabolic process | Biological Process |
| GO:0043066 | 0.04473418 | 0.999062839 | 1 | 26 | negative regulation of apoptotic process | Biological Process |
| GO:0010628 | 0.045165559 | 0.999044524 | 1 | 26 | positive regulation of gene expression | Biological Process |
| GO:0006635 | 0.04764679 | 0.998934126 | 1 | 27 | fatty acid beta-oxidation | Biological Process |
| GO:0061077 | 0.054542976 | 0.998591401 | 1 | 32 | chaperone-mediated protein folding | Biological Process |
| GO:0010087 | 0.058291067 | 0.99838891 | 1 | 32 | phloem or xylem histogenesis | Biological Process |
| GO:0051603 | 0.075915812 | 0.997224287 | 1 | 45 | proteolysis involved in cellular protein catabolic process | Biological Process |
| GO:0006629 | 0.092707758 | 0.99582047 | 1 | 53 | lipid metabolic process | Biological Process |
| GO:0045087 | 0.103490744 | 0.994759031 | 1 | 60 | innate immune response | Biological Process |
| GO:0048366 | 0.122257298 | 0.992616195 | 1 | 70 | leaf development | Biological Process |
| GO:0042787 | 0.13578059 | 0.99082619 | 1 | 82 | protein ubiquitination involved in ubiquitin-dependent protein catabolic process | Biological Process |
| GO:0016192 | 0.153815702 | 0.988126533 | 1 | 94 | vesicle-mediated transport | Biological Process |
| GO:0006886 | 0.179606182 | 0.98362477 | 1 | 107 | intracellular protein transport | Biological Process |
| GO:0009733 | 0.183079313 | 0.982954968 | 1 | 112 | response to auxin | Biological Process |
| GO:0042742 | 0.235335619 | 0.971169334 | 1 | 148 | defense response to bacterium | Biological Process |
| GO:0030154 | 0.286445319 | 0.956289251 | 1 | 187 | cell differentiation | Biological Process |
| GO:0071555 | 0.319496723 | 0.944794587 | 1 | 206 | cell wall organization | Biological Process |
| GO:0006397 | 0.328769656 | 0.94129134 | 1 | 212 | mRNA processing | Biological Process |
| GO:0016567 | 0.359443678 | 0.928793318 | 1 | 241 | protein ubiquitination | Biological Process |
| GO:0009451 | 0.491091879 | 0.857890669 | 1 | 338 | RNA modification | Biological Process |
| GO:0006351 | 0.730430581 | 0.535849291 | 2 | 1392 | transcription, DNA-templated | Biological Process |
