# Supplementary material for: Genome-wide identification and functional prediction of tobacco lncRNAs responsive to root-knot nematode stress
Source: PLoS One. 2018 Nov 14;13(11):e0204506. doi: 10.1371/journal.pone.0204506 (PMC6235259; doi:10.1371/journal.pone.0204506)
Supplement: S6 Table — (DOCX) [file pone.0204506.s008.docx]

**S6 table: QRT-PCR primers for validated lncRNAs**

|  | | |
| --- | --- | --- |
| Gene Name | Forward primer | Reverse primer |
| TCONS_00042912 | TCAACACAGACCGTAGCGAG | GAGGGCTACAACGGGCGT |
| TCONS_00152950 | GTCTTCAACCCAACGTCCAC | AACGGACAGAAGGGGTTCAA |
| TCONS_00135472 | CGCGGAAGTTTGAGGCAATA | TTCAAAGATTACCCGGGCCT |
| TCONS_00124928 | TCAGGAGGTGATGACAAGGG | ATCCAGCGTCAGGGTTTACA |
| TCONS_00103329 | CCCATCGTTCGCCCTTAATC | TCGTTCGTGTGGAGTCTTGA |
| TCONS_00074636 | ACCCTGTCCGTTCTGTTCAA | GTTAGGCTTGATGGGCATGG |
| TCONS_00199181 | GTCAGGTGGGGAGTTTAGCT | CCCTTCTGTTCCACACGAGA |
| TCONS_00132007 | ATTGTCACTACCTCCCCGTG | GGGTGACGGAGAATTAGGGT |
| TCONS_00006310 | ATTTGGTCCCAGTGGCGT | GACGGGGCAGTTATACCTCA |
| TCONS_00187326 | AGCTTTTCGAATCAAGGCGG | ACCTCAAAGTGCAGAAGTGG |
| TCONS_00132868 | CCTCTTGTCTTGTGTTTGCCA | ACGTGGACTGGAGATGTTGA |
| TCONS_00031623 | GCGCTAACCTAGATCCCACA | GCTAGTTGATTCGGCAGGTG |
| TCONS_00011888 | AGTGGATTTGATTCGCGCAG | CAACTACACCGCTCCCCTTA |
| TCONS_00239960 | CTGGCCAGCAAAACTCGTTA | AGGAAGGTTCGATTGAGGCT |
| TCONS_00060978 | AGGAACCCGCAAATATTGGA | CAAACCTGTGCTCGAAAATCAC |
|  |  |  |
